# The promotion effect of FeS_2_ on Sb_2_S_3_ bioleaching and Sb speciation transformation

**DOI:** 10.3389/fmicb.2025.1475572

**Published:** 2025-01-21

**Authors:** Xing-fu Zheng, Jin-lan Xia, Zhen-yuan Nie, Hong-peng Cao, Rui-Jia Hu, Yu-ting Liang, Hong-chang Liu

**Affiliations:** ^1^School of Minerals Processing and Bioengineering, Central South University, Changsha, China; ^2^Guangxi Academy of Sciences, Nanning, China; ^3^Key Lab of Biometallurgy of Ministry of Education of China, Central South University, Changsha, China

**Keywords:** Sb_2_S_3_, FeS_2_, bioleaching, XANES spectroscopy, electrochemistry, DFT calculations

## Abstract

Stibnite (Sb_2_S_3_) is an important but difficult to biologically leach mineral, so it is important to find a potential scheme for improving the bioleaching rate of Sb_2_S_3_. In this study, by combining experiments and first-principles density functional theory (DFT) calculations, the impact and related mechanisms of pyrite (FeS_2_) on stibnite (Sb_2_S_3_) bioleaching were studied for the first time. The bioleaching results revealed that FeS_2_ obviously improved the Sb_2_S_3_ bioleaching rate, and in the 0.5FeS_2_:0.5CuFeS_2_ system, the bioleaching rate of Sb_2_S_3_ increased from 2.23 to 24.6%, which was the best mass mixing ratio. The XPS and XANES results revealed that during the bioleaching process, Sb_2_S_3_ was transformed to Sb_2_O_3_ and Sb_2_O_5_. The electrochemical results revealed that after FeS_2_ was mixed, a FeS_2_-Sb_2_S_3_ galvanic cell formed, which promoted the electron transfer efficiency and redox reaction of Sb_2_S_3_. The DFT results show that between the Sb_2_S_3_ (0 1 0) and FeS_2_ (1 0 0) surfaces, S-Fe, S-S, S-Sb, and Sb-Fe bonds are formed, and the direction of electron transfer is from Sb_2_S_3_ to FeS_2_; the work functions for Sb_2_S_3_ after addition of FeS_2_ decrease, implying that faster electron transfer occurs; Fe(III)-6H_2_O derived from FeS_2_ adsorbs on the surface more easily than does glucose, which is the major component of the extracellular polymeric substances in bacteria, indicating that during the bioleaching process, Fe(III)-6H_2_O plays an important role; after mixing, both Fe(III)-6H_2_O and glucose adsorb on the Sb_2_S_3_ (0 1 0) surface more easily, with stronger bonds and larger adsorption energies, which are in good agreement with the experimental results.

## 1 Introduction

Antimony (Sb) plays an important role in social development and is used in storage batteries, printing industries, semiconductors, and pharmaceuticals (Awe and Sandström, 2013; Zhang et al., 2019) and is an important strategic material. Currently, via the pyrometallurgical route, Sb can be extracted from stibnite (Sb_2_S_3_), which is the most important and ubiquitous antimony ore (Biver and Shotyk, 2012; Multani et al., 2016), but such a method results in high-energy consumption and environmental pollution. In addition, with the mining of antimony ore and the decrease in high-grade antimony ores, there is a need to develop new methods to extract Sb from low-grade ores or tailings.

Bioleaching is a green, low-cost, and low-emission technology used to extract metal ions from ores (Hong et al., 2023; Zhao et al., 2020), and plenty of bioleaching research has been carried out on sulfide ore. For Sb_2_S_3_, several researchers have explored the dissolution process of Sb_2_S_3_ and reported that microorganisms play important roles in the release, migration, and transformation processes of Sb_2_S_3_ (Bagherifam et al., 2021; Loni et al., 2020), as well as the environmental processes and relevant molecular mechanisms of antimony in mining areas (Wang et al., 2020; Yang and He, 2015; Ye et al., 2020). These studies have focused mainly on the environmental effects of environmental microorganisms. The use of acidophiles to extract Au from refractory gold ores containing abundant stibnite and gudmundite has also been studied (de Carvalho et al., 2019); recently, we studied the dissolution of stibnite mediated by *Acidithiobacillus ferrooxidans* (*A. ferrooxidans*) and relevant Sb and S speciation transformations and reported that *A. ferrooxidans* can enhance the leaching process of stibnite in comparison with sterile control experiments (Wang et al., 2022). During the bioleaching process, microorganisms convert antimony through direct oxidation or indirect reduction, that is, Sb(III) is oxidized as an energy metabolism substrate to Sb(V), as shown in Equations (1)–(3), obtaining the energy required for its growth (Loni et al., 2020; Lu et al., 2018). However, the bioleaching rate is not high because of the toxicity and insolubility of Sb_2_S_3_. Therefore, it is necessary to study methods to improve the antimony leaching rate. The associated minerals significantly impact on mineral dissolution (Multani et al., 2016; Wilson et al., 2004); however, few studies have investigated about how pyrite (FeS_2_), a common natural mineral associated with Sb_2_S_3_, affects the bioleaching rate of Sb_2_S_3_ (Yan et al., 2020) and the transformation process of Sb during the bioleaching process, and the related mechanisms are still unclear.


(1) $${Sb}_{2}S_{3}\left(s\right)\overset{\;\mathrm{Microbial}\;}{\to }Sb^{3+}\left(aq\right)+S^{2-}\left(aq\right)$$

(2) $$Sb^{3+}\left(aq\right)\overset{\;\mathrm{Microbial}\;}{\to }Sb^{5+}\left(aq\right)+2e^{-}$$

(3) $$Sb^{5+}\left(aq\right)+5H_{2}O\left(l\right)\overset{\;\mathrm{Microbial}\;}{\to }{Sb}_{2}O_{5}\left(s\right)+10H^{+}\left(aq\right)+5e^{-}$$

In the present study, the effects of FeS_2_ on the fate and speciation transformation of Sb during the bioleaching of stibnite were studied via synchrotron radiation-based Sb X-ray near-edge structure (XANES) spectroscopy, and X-ray photoelectron spectroscopy (XPS) analyses, combined with density functional theory (DFT) calculations. In bioleaching bacterial strains, e.g., *Sulfobacillus thermosulfidooxidans* (Liu et al., 2024; Li, 2017), extracellular polymeric substances (EPS) are important components that are beneficial for bacterial adhesion processes (Wang et al., 2010), and glucose is the major sugar component in EPS (Gehrke et al., 1998); thus, in DFT calculations, glucose is utilized to simulate the interactions between bacteria and minerals (Zheng et al., 2020), and the Fe(III)-6H_2_O that is oxidized from FeS_2_ is also considered to simulate indirect effects during the bioleaching process (Zheng et al., 2019; Magini, 1979; Magini and Radnai, 1979). This study aims to understand the element migration mechanism in the Sb mining area, thus further identifying a potential scheme for improving the bioleaching rate of Sb_2_S_3_. To our knowledge, reports exploring the interfacial interactions for the FeS_2_-Sb_2_S_3_ bioleaching system by combining experiments and DFT calculations are rare.

## 2 Materials and methods

### 2.1 Minerals

**Figure 1:**
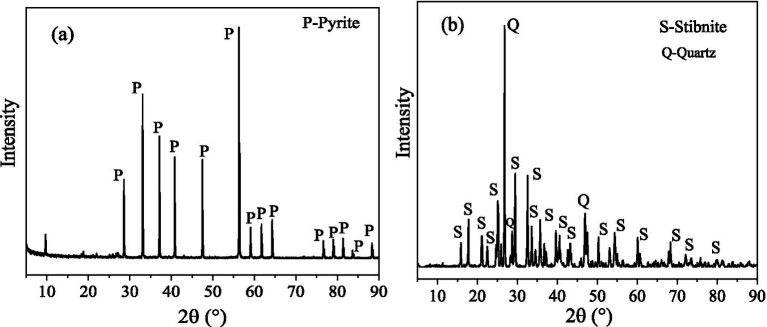
XRD results for FeS_2_ **(A)** and Sb_2_S_3_ **(B)**.

The minerals Sb_2_S_3_ and FeS_2_ were provided by the School of Minerals Processing and Bioengineering, Central South University, Changsha, China. The XRD (X-ray diffraction patterns) results in Figure 1 revealed that the pyrite is pure, and the stibnite is mainly composed of Sb_2_S_3_ and quartz. Furthermore, the composition of stibnite was determined by XRF (X-ray fluorescence), and the results (Supplementary Table S1) show that Sb, S, and Si are the main components, with a small amount of Al. The ICP (inductively coupled plasma-optical emission spectroscopy) results revealed that the contents of Sb, S, and Fe were 2:3:0. Before the bioleaching tests, the mineral samples were crushed and milled to 37–74 μm particle sizes. FeS_2_ and Sb_2_S_3_ were mixed well at weight ratios of 0:1, 0.1:0.9, 0.2:0.8, 0.3:0.7, 0.5:0.5, and 0.8:0.2.

### 2.2 Bioleaching experiments

For the bioleaching experiments, the mixed samples were used as energy substrates, and the pulp density was 1% (w/v). The bacterium, *Sulfobacillus thermosulfidooxidans* YN22 (*S. thermosulfidooxidans*), provided by the School of Minerals Processing and Bioengineering, was used; the inoculation concentration was 4 × 10^7^ cells/mL in 100 mL 9 K medium, and the pH was adjusted to 2.0. Then, the flasks were placed in a rotary shaker at 180 rpm and 45°C. The pH and ORP during the bioleaching process were determined by a pH meter (PHS-3C) and Pt electrode using a calomel electrode (Ag/AgCl) as the reference, respectively; the concentrations of Sb and Fe were determined by ICP (SPECTROBLUE FMX26, Philadelphia, PA, United States), and the concentration of [Fe^3+^] was determined by the sulfosalicylic acid method. In detail, for [Sb] and [TFe], 1 mL of solution was collected, diluted with 10% nitric acid, and preserved at −80°C until analysis; for [Fe^3+^], 1 mL of solution was collected in the anaerobic chamber and diluted with prepared anaerobic water; then, 300 μL of 10% sulfosalicylic acid solution and 300 μL of diluted solution were added into a colorimetric tube, quantified to 10 mL with distilled water, shaken well, and then the mixed solution was measured using a microplate spectrophotometer at a wavelength of 500 nm. All the experiments were conducted in triplicate.

### 2.3 Residues composition analysis

The solid leaching residues were collected after leaching for 0, 5, and 10 days, washed three times with diluted sulfuric acid (pH 2.0) and hydrochloric acid (pH 2.0), and stored at −70°C. The surface morphologies of the residues were determined by scanning electron microscopy (SEM, Nano230, FEI) coupled with energy dispersive spectroscopy (EDS). In detail, the samples were prefixed with 25% formaldehyde, dehydrated via a graded ethanol series, coated with gold nanoparticles, and introduced into the SEM chamber for observation.

The residues phase compositions were analyzed by XRD in the range of 10–90° on a Bruker D8 instrument (BrukerAXS) with Cu Kα radiation The Sb speciation of the solid residue was analyzed by X-ray photoelectron spectroscopy (XPS). Briefly, XPS spectra were collected by an X-ray photoelectron spectrometer (Thermo Scientific K-Alpha+, United Kingdom) with a voltage and current of 12 kV and 6 mA, respectively. The obtained XPS data were analyzed in CasaXPS software, and all photoelectron binding energies were referenced to the C1s adventitious contamination peak set at 284.5 eV BE. Furthermore, Sb L-edge XANES spectroscopy was performed at beamline 4B7A in the Beijing Synchrotron Radiation Facility, Beijing, China. The Sb L-edge XANES spectra were recorded in total electron yield (TEY) mode with a step size of 0.1 eV and a dwell time of 2 s at each energy at 25°C from 4.60 to 4.80 keV across the Sb L-edge. Owing to the easy oxidization of the sample surfaces, all samples and tests were performed under strict anaerobic conditions with high-purity nitrogen gas (Goh et al., 2006), and were detected under the same conditions and parameter settings. The XANES spectra were normalized to the maximum of the absorption spectrum using reference spectra with the IFEFFIT program (Ide-Ektessabi et al., 2004; Prange, 2008; Ravel and Newville, 2005).

### 2.4 Electrochemical experiments

Electrochemical measurements were performed in 9 K medium (pH 2.0) via an electrochemical working station (INTERFACE 1010E, GAMRY, America). A conventional three-electrode system was used, including a counter electrode (carbon rods), a reference electrode (Ag/AgCl), and a working electrode (mineral electrode). The working electrodes were prepared by mixing 0.3 g graphite, 1.05 g minerals, and 0.15 g solid paraffin, and then the mixture was compressed at 120 KPa for 10 min. The Tafel curves were tested from −200 to +750 mV (vs open circuit potential, OCP) with a scan rate of 1 mV/s; the EIS (electrochemical impedance spectroscopy) curves were tested in the frequency range of 10^−1^ to 10^−5^ Hz, and fitted by Gamry Echem Analyst; the forward CV (cyclic voltammetry) was scanned from −1.0 to +1.0 V, while the reversed CV from +1.0 to −1.0 V. To explore the role of bacteria, *S. thermosulfidooxidans* was added to the medium with an inoculation amount of 2*10^8^ cells/mL during the CV test. In this study, all the potentials reported were expressed vs. Ag/AgCl.

### 2.5 Computational details

The DFT calculations were performed via CASTEP (Cambridge Sequential Total Energy Package) (Segall et al., 2002) and GGA-PBE (Generalized gradient approximation-Perdew-Burke-Ernzerhof functional) (Perdew et al., 1996; Segall et al., 2002), in which only the valence electrons were considered explicitly using ultrashort pseudopotentials (Vanderbilt, 1990). Sb_2_S_3_ belongs to the space group Pmn21 (Park et al., 2010), and FeS_2_ belongs to the space group Th^6^-Pa3 (Qiu et al., 2004). After obtaining the Sb_2_S_3_ (0 1 0) surface and the FeS_2_ (1 0 0) surface, which are the most stable surfaces of the two sulfide ores (Blanchard et al., 2007; Cao et al., 2018; de Lima et al., 2011; de Oliveira et al., 2012), the supercells were built with a vacuum slab of 15 Å to avoid adjacent interlayer interactions (Fan et al., 2017). The FeS_2_-Sb_2_S_3_ interaction model was built by using building layer tools, and the calculation was performed after constraining the model size. A 3 × 3 × 1 k-point and 500 eV cutoff energy were used for the calculations. Glucose and Fe(III)-6H_2_O were optimized in a 15 × 15 × 15 Å slab. The convergence tolerances were set to a maximum displacement of 0.002 Å, a maximum force of 0.05 eV/Å, a maximum energy change of 2.0 × 10^−5^ eV/atom, and a maximum stress of 0.1 GPa, while the SCF convergence tolerance was set to 2.0 × 10^−6^ eV/atom. For all the calculations, spin polarization, dipole correction, and DFT-D correction were considered. The frontier orbitals, HOMO-LUMO, were calculated in DMol^3^. According to previous work, the calculation method above can provide reliable results for Sb_2_S_3_ and FeS_2_ (Cao et al., 2018; Cao et al., 2020; Zheng et al., 2018).

The glucose or Fe(III)-6H_2_O adsorption energies (E_ads_) can be calculated using equation 4:


(4) $$E_{\mathrm{ads}}=E_{\left(FeS2-Sb2S3-\mathrm{adsorbate}\right)}-\left(E_{FeS2-Sb2S3}+E_{\mathrm{adsorbate}}\right)$$

where E_FeS2-CuFeS2_, E_adsorbate_, and E_(FeS2-CuFeS2 - adsorbate)_ represent the total energies for the clean FeS_2_-Sb_2_S_3_ surface, the free glucose/Fe(III)-6H_2_O, and the FeS_2_-Sb_2_S_3_–adsorbate system, respectively.

## 3 Results and discussion

### 3.1 Leaching parameters

**Figure 2:**
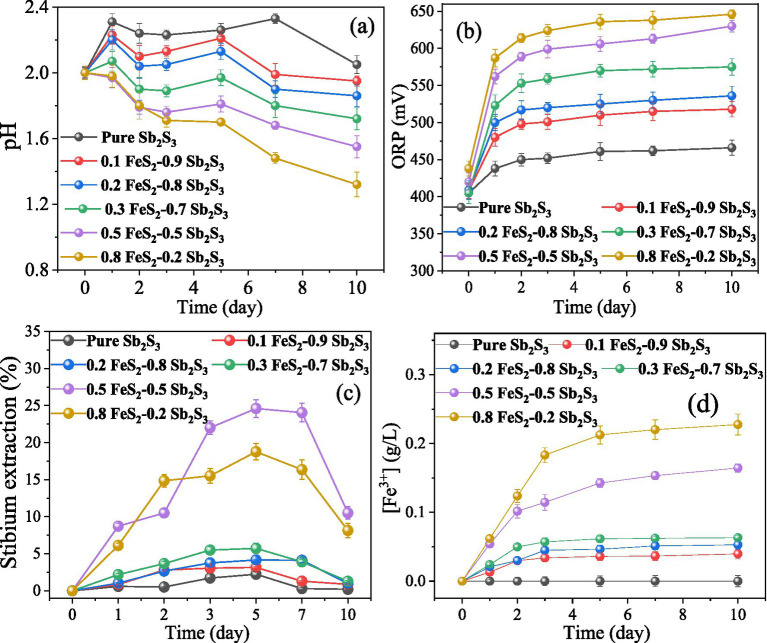
Curves for the pH **(A)**, ORP **(B)**, Sb extraction rate **(C)**, and Fe^3+^ concentration for the bioleaching of Sb_2_S_3_ in the presence of varying concentrations of FeS_2_.

The results (Figure 2) show that by adding FeS_2_, the pH decreases faster while the ORP increases faster, implying that the dissolution process of Sb_2_S_3_ may be accelerated. After leaching for 5 days, the extraction rates of Sb were approximately 2.22% (Sb_2_S_3_), 3.15% (0.1 FeS_2_: 0.9 Sb_2_S_3_), 4.1% (0.2 FeS_2_: 0.8 Sb_2_S_3_), 5.7% (0.3 FeS_2_: 0.7 Sb_2_S_3_), 24.6% (0.5 FeS_2_: 0.5 Sb_2_S_3_), and 18.7% (0.8 FeS_2_: 0.2 Sb_2_S_3_), where in the sterile control results (Supplementary Figure S1), the extraction rates of Sb were lower than 0.7%. After 10 days of leaching, the extraction rates of Sb decreased, and the reason may be the formation of secondary products that were found by the further results. In the 0.5 FeS_2_: 0.5 Sb_2_S_3_ system, the Sb dissolution rate was almost 11 times higher than that of pure Sb_2_S_3_, indicating that such a mixing ratio is the best leaching group. In addition, Figure 2D shows that Fe^3+^ occurs after the addition of FeS_2_ because FeS_2_ is oxidized by *S. thermosulfidooxidans*. In addition, Fe^3+^ attacks minerals (Jiang et al., 2019), which is called indirect action, thereby accelerating the dissolution of minerals. Notably, the concentration of Fe^3+^ is lower than that of the total Fe (Supplementary Figure S2A), which is probably because of formation of Fe^2+^ (Ubaldini et al., 2000; Zhang et al., 2019), as shown in Supplementary Figure S2B. In the next section, the 0.5 FeS_2_: 0.5 Sb_2_S_3_ mixture is analyzed further.

**Figure 3:**
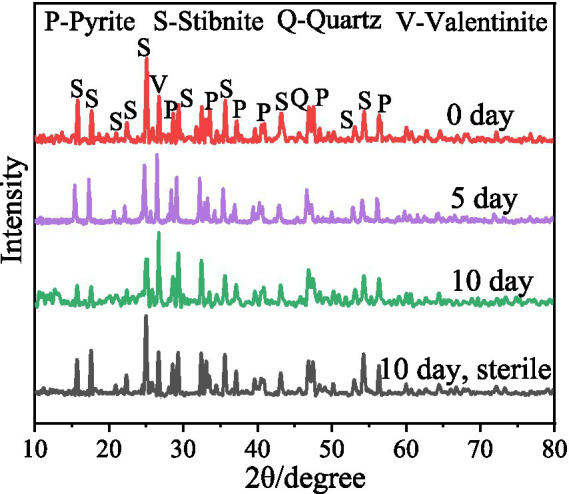
XRD of the bioleaching residues.

**Figure 4:**
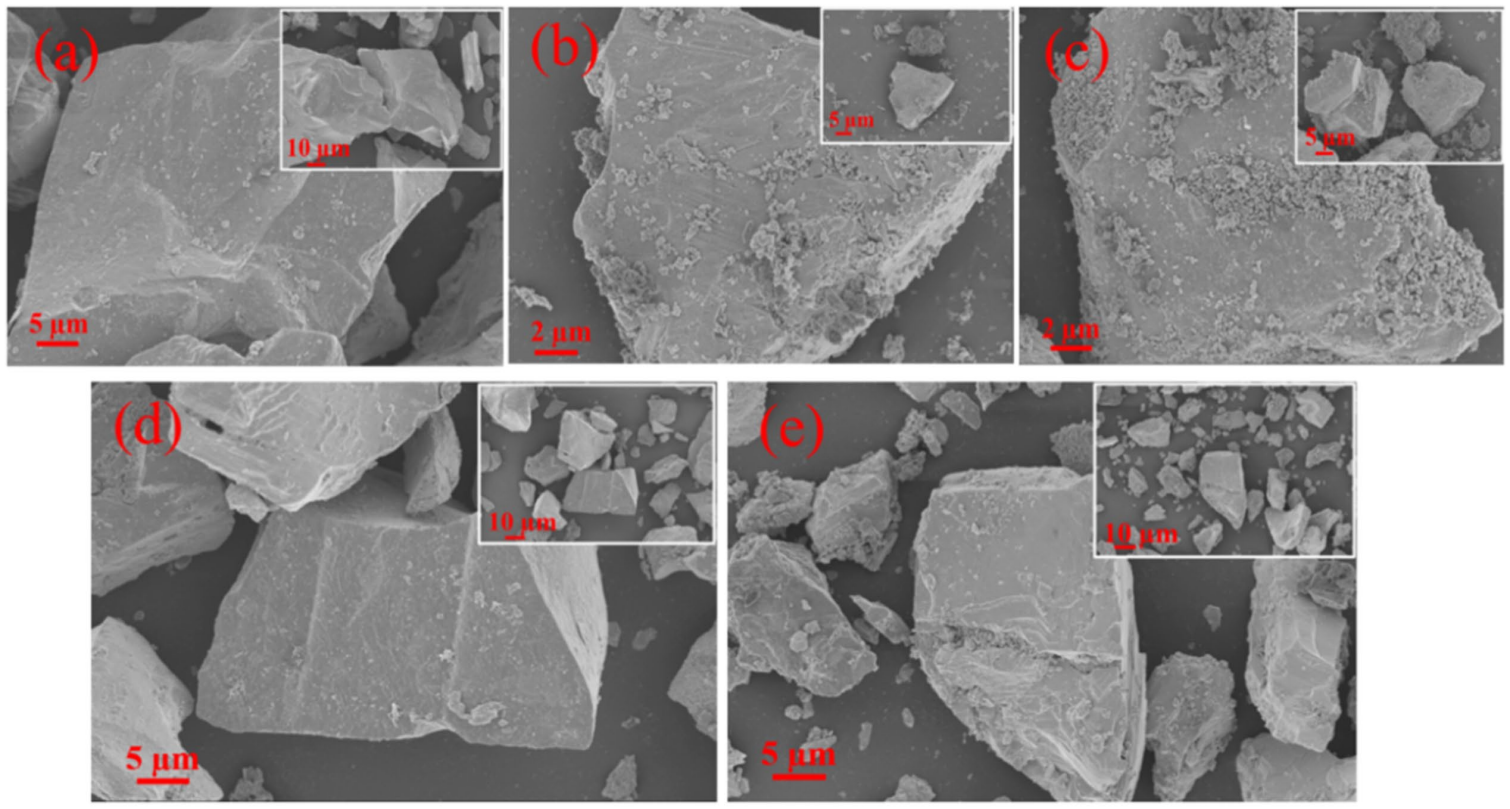
**(A–C)** SEM images of the bioleaching residues after 0, 5, and 10 days with the addition of FeS_2_; **(D,E)** SEM images of the residues after 0 and 10 days in the sterile control.

The XRD results (Figure 3) revealed that after 5 and 10 days of bioleaching, the peaks of FeS_2_ at 47.5° weakened, implying the dissolution of FeS_2_; the peak at 27° associated with Sb_2_O_3_ became stronger, and the peaks at 15.7° and 17.6° associated with Sb_2_S_3_ became weaker, indicating that Sb_2_S_3_ was transformed to Sb_2_O_3_ during bioleaching (Wang et al., 2022). Notably, in the sterile control sample, the peaks presented little or no change after 10 days of leaching. The SEM results (Figure 4; Supplementary Figure S3) show that by adding FeS_2_, the Sb_2_S_3_ surface obviously changed after 5 days of leaching with obvious secondary products, whereas the changes in pure Sb_2_S_3_ (Supplementary Figure S4) were negligible, confirming the promoting effect of FeS_2_, and the source of secondary minerals was the dissolution of some FeS_2_ (Yang et al., 2016). In addition, in the sterile control sample (Figures 4D,E), the mineral surface changed little, indicating that bacteria play an important role in the oxidation of minerals.

**Figure 5:**
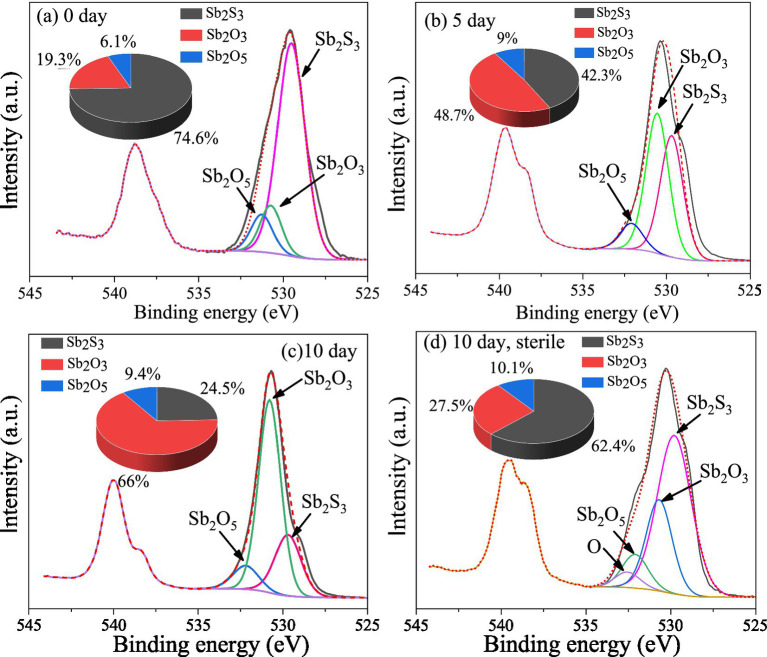
XPS spectra of Sb on the surface of the bioleaching residues.

The XPS results (Figure 5) show that after 5 days of bioleaching, part of Sb_2_S_3_ (529.5 eV) (Morgan et al., 1973) was transformed to Sb_2_O_3_ (530.5 eV, 48.7%) and Sb_2_O_5_ (532.1 eV, 9%), and the proportion of Sb_2_S_3_ decreased from 74.6 to 42.3%; as the leaching time increased to 10 days, the proportion of Sb_2_O_3_ increased to 66%, and that of Sb_2_O_5_ increased to 9.4%, whereas the proportion of Sb_2_S_3_ decreased to 24.5%; in the sterile controlled experiment (Figure 5D), the proportion of Sb_2_S_3_ decreased to 62.4% after 10 days of leaching, which was much slower.

**Figure 6:**
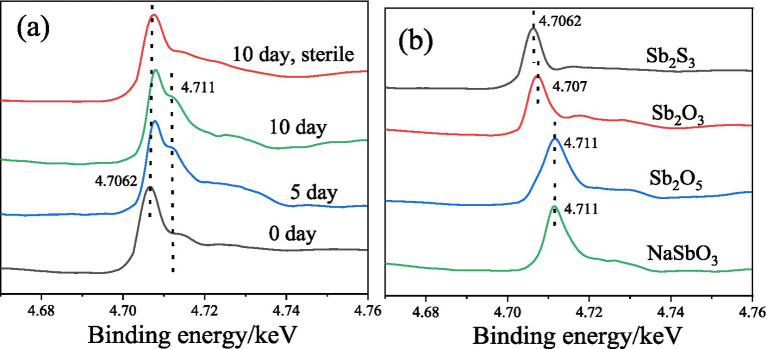
Sb L-edge XANES spectra of the residues during bioleaching **(A)** and the reference materials **(B)**.

The Sb L-edge XANES spectra (Figure 6) show that after 5 and 10 days of bioleaching, the peak shifts from 4.7062 to 4.707 keV, indicating the conversion of Sb_2_S_3_ to Sb_2_O_3_, and the peak at 4.711 keV increases with increasing bioleaching time, which indicates the conversion of Sb(III) to Sb(V), similar to the XPS results. In addition, in the sterile controlled experiment, after 10 days, no or little Sb(III) was converted to Sb(V), confirming the important role of bacteria.

### 3.2 Electrochemical analyses

**Figure 7:**
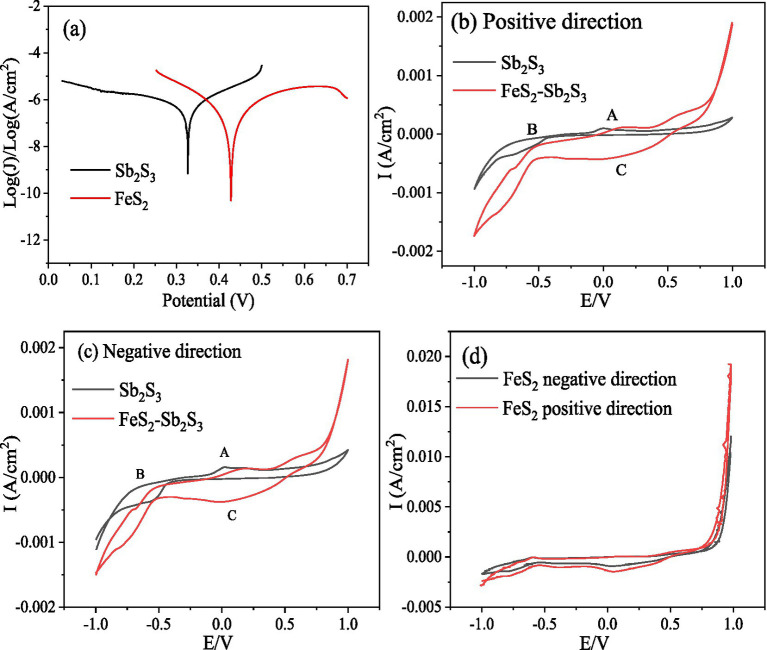
Tafel and CV curves for FeS_2_, Sb_2_S_3_ and FeS_2_-Sb_2_S_3_.

For mineral-mineral interactions, a galvanic effect may occur when two minerals have different corrosion potentials; however, minerals with higher potentials can act as cathodes and the other minerals with lower potential can act as anodes electrode (Ekmekqi and Demirel, 1997). The lower the value of the corrosion potential is, the easier it is for the mineral to corrode. Tafel tests were performed to analyze the mineral corrosion kinetics. The Tafel results (Figure 7A) show that the corrosion potentials (vs. Ag/AgCl) for FeS_2_ and Sb_2_S_3_ are 428 mV and 327 mV, respectively; thus in a FeS_2_-Sb_2_S_3_ system, the galvanic effect occurs, and Sb_2_S_3_ is the anode electrode, implying that the leaching rate of Sb_2_S_3_ is promoted (Zheng et al., 2021).

Among electrochemical methods, CV is widely used due to its simple operation and effective results for the interpretation of electrochemical reactions. The results in Figure 7 show that the oxidation peaks of Sb_2_S_3_ and the corresponding reduction peaks are not symmetrical, indicating that the redox reaction on the Sb_2_S_3_ surface is an irreversible process (Córdoba et al., 2009). Figures 7B,C shows that the CV peaks of FeS_2_-Sb_2_S_3_ are similar to those of Sb_2_S_3_ (A1, B1, and C1), implying that Sb_2_S_3_ reacts preferentially during the electrochemical process. The current density is greater after addition of FeS_2_, indicating an increase in the Sb_2_S_3_ reaction.

To investigate the effects of bacteria on the dissolution of Sb_2_S_3_, the effects of CV with *S. thermosulfidooxidans* were analyzed. Supplementary Figure S5 shows that after adding bacteria (2*10^8^ cells/ml), the current density increased further, confirming that the bacteria can obviously enhance the leaching rate of Sb_2_S_3_. During the bioleaching process, some of the FeS_2_ is oxidized by bacteria to produce Fe^3+^ (Yang et al., 2016), so the effect of Fe^3+^ was also studied, and the results (Supplementary Figure S6) revealed that after adding Fe^3+^ (0.15 g/L), the current density also increased, indicating that Fe^3+^ can also enhance the leaching rate of Sb_2_S_3_.

**Figure 8:**
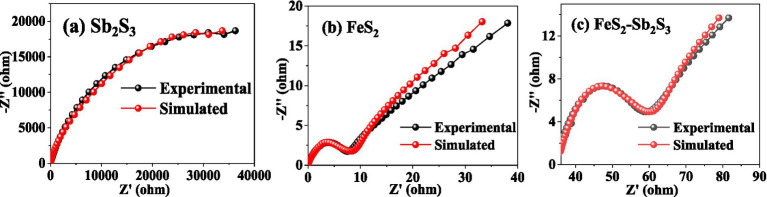
Nyquist plots for Sb_2_S_3_ **(A)**, FeS_2_ **(B)**, and FeS_2_-Sb_2_S_3_ **(C)**.

The electron transfer efficiency of minerals was analyzed via EIS. The data obtained were analyzed by fitting the impedance data to an appropriate equivalent circuit as Rs(Q1(R1Q2)) (Bevilaqua et al., 2009; Supplementary Figure S7). In the equivalent circuit, Rs, R1, and Q1 represent the solution resistance, ion exchange impedance, and constant phase element, respectively. Q1 is connected to the electrode interface. Q2 represents a Warburg element, and is related to the electrode/electrolyte interface diffusion process (Zeng et al., 2020). Figure 8 shows that after adding FeS_2_, the curve radius decreases; the results in Table 1 show that R1 for FeS_2_-Sb_2_S_3_ (30.71 *Ω*·cm^−2^) is much smaller than that for Sb_2_S_3_ (24,300 Ω·cm^−2^). Both results indicate that after mixing, the ion exchange resistance on the mineral surface decreases; in other words, after Sb_2_S_3_ mixed with FeS_2_, the leaching system has a relatively high electron transfer efficiency, which significantly promotes the bioleaching rate.

**Table 1:** Fitting results obtained via the circuit Rs(Q1(R1Q2)) for Sb_2_S_3_, FeS_2,_ and FeS_2_-Sb_2_S_3_.

	Rs Ω·cm^−2^	R1 *Ω*·cm^−2^	Q1-T S·s^a^·cm^−2^	Q1-P S·s^a^·cm^−2^	Q2 S·s^1/2^·cm^−2^
Sb_2_S_3_	16.73	24,300	1.781*10^−5^	0.817	6.820*10^−5^
FeS_2_	16.73	10	9.25*10^−4^	0.753	9.027*10^−2^
FeS_2_-Sb_2_S_3_	16.73	30.71	8.59*10^−4^	0.631	7.872*10^−2^

### 3.3 Computational results

#### 3.3.1 Electronic structure

**Figure 9:**
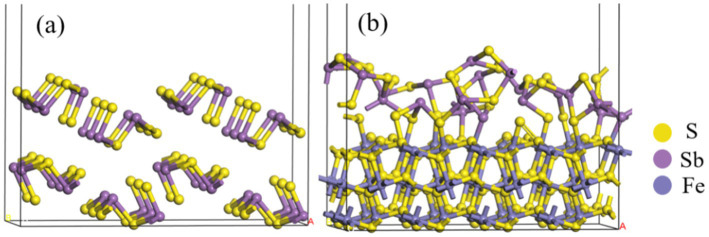
Optimized configurations of Sb_2_S_3_ **(A)**, and FeS_2_-Sb_2_S_3_ **(B)**.

The models of Sb_2_S_3_ and FeS_2_-Sb_2_S_3_ are shown in Figure 9, and the corresponding atomic numbers used are shown in Supplementary Figure S8. The results in Figure 9 show that after adding FeS_2_, the surface structure of Sb_2_S_3_ gradually became disordered, which was conducive to the dissolution of Sb_2_S_3_. Table 2 shows that after adding FeS_2_, the Hirshfeld charge value of FeS_2_ decreases from 0 to −0.77, whereas the charge value of Sb_2_S_3_ increases from 0 to 0.77, indicating that the direction of electron transfer is from Sb_2_S_3_ to FeS_2_.

**Table 2:** Hirshfeld charge analysis of FeS_2_-Sb_2_S_3_.

	Charge/e Before optimization	Charge/e After optimization
FeS_2_	0	−0.77
Sb_2_S_3_	0	0.77

The bond lengths between FeS_2_ and Sb_2_S_3_ are shown in Table 3. In the FeS_2_-Sb_2_S_3_ galvanic cell, S-Fe, S-S, Sb-Fe, and S-Sb bonds formed at the interface, and the number of S-Fe bonds was greater than that of the other materials. S3-S23 is the shortest bond (2.128 Å), whereas Sb12-S48 is the longest bond (2.619 Å). The Mulliken bond population results show that the S2-Fe2 bond has stronger covalent interactions, and that the Sb5-S32 bond has stronger ionic interactions.

**Table 3:** Mulliken bond population analysis of FeS_2_-Sb_2_S_3_.

Bond	Bond Lengths (Å)	Populations
S2-Fe2	2.354	0.44
S3-S23	2.128	0.25
S4-Fe4	2.307	0.44
S6-Fe5	2.267	0.42
S7-Fe6	2.491	0.32
Sb4-Fe8	2.546	0.14
Sb5-S32	2.606	0.10
S12-Fe11	2.306	0.26
S13-Fe12	2.334	0.41
S15-Fe13	2.323	0.42
S16-Fe14	2.306	0.26
Sb11-Fe16	2.547	0.21
Sb12-S48	2.619	0.20

**Figure 10:**
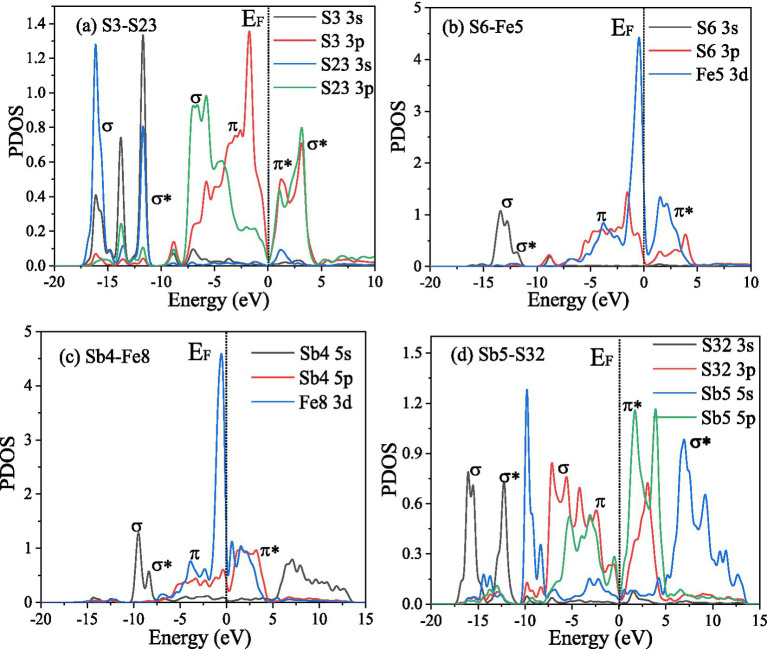
PDOS of the bonds between the FeS_2_ and Sb_2_S_3_ surfaces.

The PDOS of the S3-S23, S6-Fe5, Sb4-Fe8, and Sb6-S32 bonds were analyzed further. Figure 10 shows that in the S3-S23 bond, from −20 eV to 10 eV, the main peaks belong to *σ*(2 s), σ*(2 s), σ(2p), *π*(2p), π*(2p), and σ*(2p) bonds, and the maximum overlap area between S 3p ranges from −10 eV to 5 eV, implying that σ(2p) and π(2p) are the main covalent interactions; in the S6-Fe5 bond, from −15 eV to 5 eV, the main peaks belong to σ(s-d), σ*(s-d), π(p-d), and π*(p-d) bonds, and the maximum overlap area between S 3p and Fe 3d ranges from −10 eV to 5 eV, implying that π(p-d) are the main covalent interactions, and the same result can also be obtained in the Sb4-Fe8 bond; in the Sb6-S32 bond, the antibonding function from 0 eV to 15 eV is strong, implying weak covalent interactions, similar to the population results.

**Figure 11:**
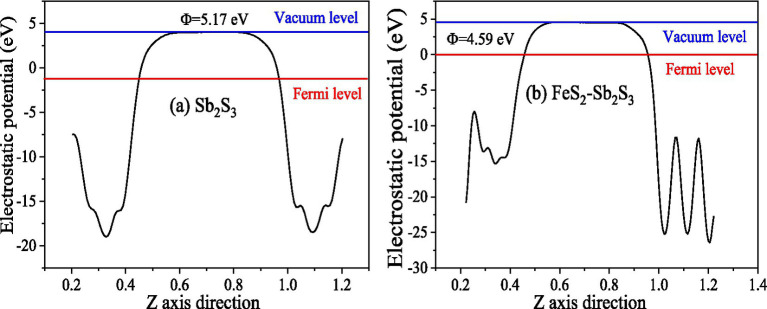
Work functions of Sb_2_S_3_ **(A)**, and FeS_2_-Sb_2_S_3_ **(B)**.

The work functions (Figure 11) for Sb_2_S_3_ and FeS_2_-Sb_2_S_3_ were calculated to be 5.17 eV, and 4.59 eV, respectively, implying that the electron transfer efficiency becomes faster after mixing, which agrees with the EIS results above.

#### 3.3.2 Adsorption configurations and energies

In the theory of frontier orbitals, HOMO (highest occupied molecular orbital) can donate electrons, and LUMO (lowest unoccupied molecular orbital) can accept electrons (Sauer and Sustmann, 1980). According to previous studies (Sauer and Sustmann, 1980; Zheng et al., 2018), a smaller HOMO-LUMO energy difference absolute value implies a more beneficial interaction. The results in Table 4 shows that ΔE_2_ is lower than ΔE_1_, indicating that the interaction mainly occurs between the HOMO of glucose and LUMO of FeS_2_-Sb_2_S_3_ rather than the HOMO of FeS_2_-Sb_2_S_3_ and the LUMO of glucose; the results also show that ΔE_4_ is lower than ΔE_3_, implying a beneficial interaction between the HOMO of Fe(III)-6H_2_O and LUMO of FeS_2_-Sb_2_S_3_.

**Table 4:** Frontier orbital energies of FeS_2_-CuFeS_2_ and glucose/Fe(III)-6H_2_O.

	HOMO	LUMO	ΔE1	ΔE2	ΔE3	ΔE4
0.1496	0.1496	0.1496	0.1496	0.1496	0.1496	0.1496
0.1496	0.1496	0.1496	0.1496	0.1496	0.1496	0.1496
0.1496	0.1496	0.1496	0.1496	0.1496	0.1496	0.1496

ΔE1 = |E (HOMOFeS2-Sb2S3)-E (LUMOglucose)|; ΔE2 = |E (HOMOglucose)-E (LUMOFeS2-Sb2S3)|; ΔE3 = |E (HOMOFeS2-Sb2S3)-E (LUMOFe(III)-6H2O)|; ΔE4 = |E (HOMOFe(III)-6H2O)-E (LUMOFeS2-Sb2S3)|.ΔE1 = |E (HOMOFeS2-Sb2S3)-E (LUMOglucose)|; ΔE2 = |E (HOMOglucose)-E (LUMOFeS2-Sb2S3)|; ΔE3 = |E (HOMOFeS2-Sb2S3)-E (LUMOFe(III)-6H2O)|; ΔE4 = |E (HOMOFe(III)-6H2O)-E (LUMOFeS2-Sb2S3)|.ΔE1 = |E (HOMOFeS2-Sb2S3)-E (LUMOglucose)|; ΔE2 = |E (HOMOglucose)-E (LUMOFeS2-Sb2S3)|; ΔE3 = |E (HOMOFeS2-Sb2S3)-E (LUMOFe(III)-6H2O)|; ΔE4 = |E (HOMOFe(III)-6H2O)-E (LUMOFeS2-Sb2S3)|.ΔE1 = |E (HOMOFeS2-Sb2S3)-E (LUMOglucose)|; ΔE2 = |E (HOMOglucose)-E (LUMOFeS2-Sb2S3)|; ΔE3 = |E (HOMOFeS2-Sb2S3)-E (LUMOFe(III)-6H2O)|; ΔE4 = |E (HOMOFe(III)-6H2O)-E (LUMOFeS2-Sb2S3)|.ΔE1 = |E (HOMOFeS2-Sb2S3)-E (LUMOglucose)|; ΔE2 = |E (HOMOglucose)-E (LUMOFeS2-Sb2S3)|; ΔE3 = |E (HOMOFeS2-Sb2S3)-E (LUMOFe(III)-6H2O)|; ΔE4 = |E (HOMOFe(III)-6H2O)-E (LUMOFeS2-Sb2S3)|.ΔE1 = |E (HOMOFeS2-Sb2S3)-E (LUMOglucose)|; ΔE2 = |E (HOMOglucose)-E (LUMOFeS2-Sb2S3)|; ΔE3 = |E (HOMOFeS2-Sb2S3)-E (LUMOFe(III)-6H2O)|; ΔE4 = |E (HOMOFe(III)-6H2O)-E (LUMOFeS2-Sb2S3)|.ΔE1 = |E (HOMOFeS2-Sb2S3)-E (LUMOglucose)|; ΔE2 = |E (HOMOglucose)-E (LUMOFeS2-Sb2S3)|; ΔE3 = |E (HOMOFeS2-Sb2S3)-E (LUMOFe(III)-6H2O)|; ΔE4 = |E (HOMOFe(III)-6H2O)-E (LUMOFeS2-Sb2S3)|.ΔE1 = |E (HOMOFeS2-Sb2S3)-E (LUMOglucose)|; ΔE2 = |E (HOMOglucose)-E (LUMOFeS2-Sb2S3)|; ΔE3 = |E (HOMOFeS2-Sb2S3)-E (LUMOFe(III)-6H2O)|; ΔE4 = |E (HOMOFe(III)-6H2O)-E (LUMOFeS2-Sb2S3)|.ΔE1 = |E (HOMOFeS2-Sb2S3)-E (LUMOglucose)|; ΔE2 = |E (HOMOglucose)-E (LUMOFeS2-Sb2S3)|; ΔE3 = |E (HOMOFeS2-Sb2S3)-E (LUMOFe(III)-6H2O)|; ΔE4 = |E (HOMOFe(III)-6H2O)-E (LUMOFeS2-Sb2S3)|._ΔE1 = |E (HOMOFeS2-Sb2S3)-E (LUMOglucose)|; ΔE2 = |E (HOMOglucose)-E (LUMOFeS2-Sb2S3)|; ΔE3 = |E (HOMOFeS2-Sb2S3)-E (LUMOFe(III)-6H2O)|; ΔE4 = |E (HOMOFe(III)-6H2O)-E (LUMOFeS2-Sb2S3)|.ΔE1 = |E (HOMOFeS2-Sb2S3)-E (LUMOglucose)|; ΔE2 = |E (HOMOglucose)-E (LUMOFeS2-Sb2S3)|; ΔE3 = |E (HOMOFeS2-Sb2S3)-E (LUMOFe(III)-6H2O)|; ΔE4 = |E (HOMOFe(III)-6H2O)-E (LUMOFeS2-Sb2S3)|.ΔE1 = |E (HOMOFeS2-Sb2S3)-E (LUMOglucose)|; ΔE2 = |E (HOMOglucose)-E (LUMOFeS2-Sb2S3)|; ΔE3 = |E (HOMOFeS2-Sb2S3)-E (LUMOFe(III)-6H2O)|; ΔE4 = |E (HOMOFe(III)-6H2O)-E (LUMOFeS2-Sb2S3)|.ΔE1 = |E (HOMOFeS2-Sb2S3)-E (LUMOglucose)|; ΔE2 = |E (HOMOglucose)-E (LUMOFeS2-Sb2S3)|; ΔE3 = |E (HOMOFeS2-Sb2S3)-E (LUMOFe(III)-6H2O)|; ΔE4 = |E (HOMOFe(III)-6H2O)-E (LUMOFeS2-Sb2S3)|.ΔE1 = |E (HOMOFeS2-Sb2S3)-E (LUMOglucose)|; ΔE2 = |E (HOMOglucose)-E (LUMOFeS2-Sb2S3)|; ΔE3 = |E (HOMOFeS2-Sb2S3)-E (LUMOFe(III)-6H2O)|; ΔE4 = |E (HOMOFe(III)-6H2O)-E (LUMOFeS2-Sb2S3)|.ΔE1 = |E (HOMOFeS2-Sb2S3)-E (LUMOglucose)|; ΔE2 = |E (HOMOglucose)-E (LUMOFeS2-Sb2S3)|; ΔE3 = |E (HOMOFeS2-Sb2S3)-E (LUMOFe(III)-6H2O)|; ΔE4 = |E (HOMOFe(III)-6H2O)-E (LUMOFeS2-Sb2S3)|.ΔE1 = |E (HOMOFeS2-Sb2S3)-E (LUMOglucose)|; ΔE2 = |E (HOMOglucose)-E (LUMOFeS2-Sb2S3)|; ΔE3 = |E (HOMOFeS2-Sb2S3)-E (LUMOFe(III)-6H2O)|; ΔE4 = |E (HOMOFe(III)-6H2O)-E (LUMOFeS2-Sb2S3)|.ΔE1 = |E (HOMOFeS2-Sb2S3)-E (LUMOglucose)|; ΔE2 = |E (HOMOglucose)-E (LUMOFeS2-Sb2S3)|; ΔE3 = |E (HOMOFeS2-Sb2S3)-E (LUMOFe(III)-6H2O)|; ΔE4 = |E (HOMOFe(III)-6H2O)-E (LUMOFeS2-Sb2S3)|.ΔE1 = |E (HOMOFeS2-Sb2S3)-E (LUMOglucose)|; ΔE2 = |E (HOMOglucose)-E (LUMOFeS2-Sb2S3)|; ΔE3 = |E (HOMOFeS2-Sb2S3)-E (LUMOFe(III)-6H2O)|; ΔE4 = |E (HOMOFe(III)-6H2O)-E (LUMOFeS2-Sb2S3)|.ΔE1 = |E (HOMOFeS2-Sb2S3)-E (LUMOglucose)|; ΔE2 = |E (HOMOglucose)-E (LUMOFeS2-Sb2S3)|; ΔE3 = |E (HOMOFeS2-Sb2S3)-E (LUMOFe(III)-6H2O)|; ΔE4 = |E (HOMOFe(III)-6H2O)-E (LUMOFeS2-Sb2S3)|._ΔE1 = |E (HOMOFeS2-Sb2S3)-E (LUMOglucose)|; ΔE2 = |E (HOMOglucose)-E (LUMOFeS2-Sb2S3)|; ΔE3 = |E (HOMOFeS2-Sb2S3)-E (LUMOFe(III)-6H2O)|; ΔE4 = |E (HOMOFe(III)-6H2O)-E (LUMOFeS2-Sb2S3)|.ΔE1 = |E (HOMOFeS2-Sb2S3)-E (LUMOglucose)|; ΔE2 = |E (HOMOglucose)-E (LUMOFeS2-Sb2S3)|; ΔE3 = |E (HOMOFeS2-Sb2S3)-E (LUMOFe(III)-6H2O)|; ΔE4 = |E (HOMOFe(III)-6H2O)-E (LUMOFeS2-Sb2S3)|.ΔE1 = |E (HOMOFeS2-Sb2S3)-E (LUMOglucose)|; ΔE2 = |E (HOMOglucose)-E (LUMOFeS2-Sb2S3)|; ΔE3 = |E (HOMOFeS2-Sb2S3)-E (LUMOFe(III)-6H2O)|; ΔE4 = |E (HOMOFe(III)-6H2O)-E (LUMOFeS2-Sb2S3)|.ΔE1 = |E (HOMOFeS2-Sb2S3)-E (LUMOglucose)|; ΔE2 = |E (HOMOglucose)-E (LUMOFeS2-Sb2S3)|; ΔE3 = |E (HOMOFeS2-Sb2S3)-E (LUMOFe(III)-6H2O)|; ΔE4 = |E (HOMOFe(III)-6H2O)-E (LUMOFeS2-Sb2S3)|.ΔE1 = |E (HOMOFeS2-Sb2S3)-E (LUMOglucose)|; ΔE2 = |E (HOMOglucose)-E (LUMOFeS2-Sb2S3)|; ΔE3 = |E (HOMOFeS2-Sb2S3)-E (LUMOFe(III)-6H2O)|; ΔE4 = |E (HOMOFe(III)-6H2O)-E (LUMOFeS2-Sb2S3)|.ΔE1 = |E (HOMOFeS2-Sb2S3)-E (LUMOglucose)|; ΔE2 = |E (HOMOglucose)-E (LUMOFeS2-Sb2S3)|; ΔE3 = |E (HOMOFeS2-Sb2S3)-E (LUMOFe(III)-6H2O)|; ΔE4 = |E (HOMOFe(III)-6H2O)-E (LUMOFeS2-Sb2S3)|.ΔE1 = |E (HOMOFeS2-Sb2S3)-E (LUMOglucose)|; ΔE2 = |E (HOMOglucose)-E (LUMOFeS2-Sb2S3)|; ΔE3 = |E (HOMOFeS2-Sb2S3)-E (LUMOFe(III)-6H2O)|; ΔE4 = |E (HOMOFe(III)-6H2O)-E (LUMOFeS2-Sb2S3)|.ΔE1 = |E (HOMOFeS2-Sb2S3)-E (LUMOglucose)|; ΔE2 = |E (HOMOglucose)-E (LUMOFeS2-Sb2S3)|; ΔE3 = |E (HOMOFeS2-Sb2S3)-E (LUMOFe(III)-6H2O)|; ΔE4 = |E (HOMOFe(III)-6H2O)-E (LUMOFeS2-Sb2S3)|.ΔE1 = |E (HOMOFeS2-Sb2S3)-E (LUMOglucose)|; ΔE2 = |E (HOMOglucose)-E (LUMOFeS2-Sb2S3)|; ΔE3 = |E (HOMOFeS2-Sb2S3)-E (LUMOFe(III)-6H2O)|; ΔE4 = |E (HOMOFe(III)-6H2O)-E (LUMOFeS2-Sb2S3)|._ΔE1 = |E (HOMOFeS2-Sb2S3)-E (LUMOglucose)|; ΔE2 = |E (HOMOglucose)-E (LUMOFeS2-Sb2S3)|; ΔE3 = |E (HOMOFeS2-Sb2S3)-E (LUMOFe(III)-6H2O)|; ΔE4 = |E (HOMOFe(III)-6H2O)-E (LUMOFeS2-Sb2S3)|.ΔE1 = |E (HOMOFeS2-Sb2S3)-E (LUMOglucose)|; ΔE2 = |E (HOMOglucose)-E (LUMOFeS2-Sb2S3)|; ΔE3 = |E (HOMOFeS2-Sb2S3)-E (LUMOFe(III)-6H2O)|; ΔE4 = |E (HOMOFe(III)-6H2O)-E (LUMOFeS2-Sb2S3)|.ΔE1 = |E (HOMOFeS2-Sb2S3)-E (LUMOglucose)|; ΔE2 = |E (HOMOglucose)-E (LUMOFeS2-Sb2S3)|; ΔE3 = |E (HOMOFeS2-Sb2S3)-E (LUMOFe(III)-6H2O)|; ΔE4 = |E (HOMOFe(III)-6H2O)-E (LUMOFeS2-Sb2S3)|.ΔE1 = |E (HOMOFeS2-Sb2S3)-E (LUMOglucose)|; ΔE2 = |E (HOMOglucose)-E (LUMOFeS2-Sb2S3)|; ΔE3 = |E (HOMOFeS2-Sb2S3)-E (LUMOFe(III)-6H2O)|; ΔE4 = |E (HOMOFe(III)-6H2O)-E (LUMOFeS2-Sb2S3)|.ΔE1 = |E (HOMOFeS2-Sb2S3)-E (LUMOglucose)|; ΔE2 = |E (HOMOglucose)-E (LUMOFeS2-Sb2S3)|; ΔE3 = |E (HOMOFeS2-Sb2S3)-E (LUMOFe(III)-6H2O)|; ΔE4 = |E (HOMOFe(III)-6H2O)-E (LUMOFeS2-Sb2S3)|.ΔE1 = |E (HOMOFeS2-Sb2S3)-E (LUMOglucose)|; ΔE2 = |E (HOMOglucose)-E (LUMOFeS2-Sb2S3)|; ΔE3 = |E (HOMOFeS2-Sb2S3)-E (LUMOFe(III)-6H2O)|; ΔE4 = |E (HOMOFe(III)-6H2O)-E (LUMOFeS2-Sb2S3)|.ΔE1 = |E (HOMOFeS2-Sb2S3)-E (LUMOglucose)|; ΔE2 = |E (HOMOglucose)-E (LUMOFeS2-Sb2S3)|; ΔE3 = |E (HOMOFeS2-Sb2S3)-E (LUMOFe(III)-6H2O)|; ΔE4 = |E (HOMOFe(III)-6H2O)-E (LUMOFeS2-Sb2S3)|._ΔE1 = |E (HOMOFeS2-Sb2S3)-E (LUMOglucose)|; ΔE2 = |E (HOMOglucose)-E (LUMOFeS2-Sb2S3)|; ΔE3 = |E (HOMOFeS2-Sb2S3)-E (LUMOFe(III)-6H2O)|; ΔE4 = |E (HOMOFe(III)-6H2O)-E (LUMOFeS2-Sb2S3)|.ΔE1 = |E (HOMOFeS2-Sb2S3)-E (LUMOglucose)|; ΔE2 = |E (HOMOglucose)-E (LUMOFeS2-Sb2S3)|; ΔE3 = |E (HOMOFeS2-Sb2S3)-E (LUMOFe(III)-6H2O)|; ΔE4 = |E (HOMOFe(III)-6H2O)-E (LUMOFeS2-Sb2S3)|.ΔE1 = |E (HOMOFeS2-Sb2S3)-E (LUMOglucose)|; ΔE2 = |E (HOMOglucose)-E (LUMOFeS2-Sb2S3)|; ΔE3 = |E (HOMOFeS2-Sb2S3)-E (LUMOFe(III)-6H2O)|; ΔE4 = |E (HOMOFe(III)-6H2O)-E (LUMOFeS2-Sb2S3)|.ΔE1 = |E (HOMOFeS2-Sb2S3)-E (LUMOglucose)|; ΔE2 = |E (HOMOglucose)-E (LUMOFeS2-Sb2S3)|; ΔE3 = |E (HOMOFeS2-Sb2S3)-E (LUMOFe(III)-6H2O)|; ΔE4 = |E (HOMOFe(III)-6H2O)-E (LUMOFeS2-Sb2S3)|.ΔE1 = |E (HOMOFeS2-Sb2S3)-E (LUMOglucose)|; ΔE2 = |E (HOMOglucose)-E (LUMOFeS2-Sb2S3)|; ΔE3 = |E (HOMOFeS2-Sb2S3)-E (LUMOFe(III)-6H2O)|; ΔE4 = |E (HOMOFe(III)-6H2O)-E (LUMOFeS2-Sb2S3)|.ΔE1 = |E (HOMOFeS2-Sb2S3)-E (LUMOglucose)|; ΔE2 = |E (HOMOglucose)-E (LUMOFeS2-Sb2S3)|; ΔE3 = |E (HOMOFeS2-Sb2S3)-E (LUMOFe(III)-6H2O)|; ΔE4 = |E (HOMOFe(III)-6H2O)-E (LUMOFeS2-Sb2S3)|.ΔE1 = |E (HOMOFeS2-Sb2S3)-E (LUMOglucose)|; ΔE2 = |E (HOMOglucose)-E (LUMOFeS2-Sb2S3)|; ΔE3 = |E (HOMOFeS2-Sb2S3)-E (LUMOFe(III)-6H2O)|; ΔE4 = |E (HOMOFe(III)-6H2O)-E (LUMOFeS2-Sb2S3)|.ΔE1 = |E (HOMOFeS2-Sb2S3)-E (LUMOglucose)|; ΔE2 = |E (HOMOglucose)-E (LUMOFeS2-Sb2S3)|; ΔE3 = |E (HOMOFeS2-Sb2S3)-E (LUMOFe(III)-6H2O)|; ΔE4 = |E (HOMOFe(III)-6H2O)-E (LUMOFeS2-Sb2S3)|.ΔE1 = |E (HOMOFeS2-Sb2S3)-E (LUMOglucose)|; ΔE2 = |E (HOMOglucose)-E (LUMOFeS2-Sb2S3)|; ΔE3 = |E (HOMOFeS2-Sb2S3)-E (LUMOFe(III)-6H2O)|; ΔE4 = |E (HOMOFe(III)-6H2O)-E (LUMOFeS2-Sb2S3)|.ΔE1 = |E (HOMOFeS2-Sb2S3)-E (LUMOglucose)|; ΔE2 = |E (HOMOglucose)-E (LUMOFeS2-Sb2S3)|; ΔE3 = |E (HOMOFeS2-Sb2S3)-E (LUMOFe(III)-6H2O)|; ΔE4 = |E (HOMOFe(III)-6H2O)-E (LUMOFeS2-Sb2S3)|.ΔE1 = |E (HOMOFeS2-Sb2S3)-E (LUMOglucose)|; ΔE2 = |E (HOMOglucose)-E (LUMOFeS2-Sb2S3)|; ΔE3 = |E (HOMOFeS2-Sb2S3)-E (LUMOFe(III)-6H2O)|; ΔE4 = |E (HOMOFe(III)-6H2O)-E (LUMOFeS2-Sb2S3)|._ΔE1 = |E (HOMOFeS2-Sb2S3)-E (LUMOglucose)|; ΔE2 = |E (HOMOglucose)-E (LUMOFeS2-Sb2S3)|; ΔE3 = |E (HOMOFeS2-Sb2S3)-E (LUMOFe(III)-6H2O)|; ΔE4 = |E (HOMOFe(III)-6H2O)-E (LUMOFeS2-Sb2S3)|.ΔE1 = |E (HOMOFeS2-Sb2S3)-E (LUMOglucose)|; ΔE2 = |E (HOMOglucose)-E (LUMOFeS2-Sb2S3)|; ΔE3 = |E (HOMOFeS2-Sb2S3)-E (LUMOFe(III)-6H2O)|; ΔE4 = |E (HOMOFe(III)-6H2O)-E (LUMOFeS2-Sb2S3)|.ΔE1 = |E (HOMOFeS2-Sb2S3)-E (LUMOglucose)|; ΔE2 = |E (HOMOglucose)-E (LUMOFeS2-Sb2S3)|; ΔE3 = |E (HOMOFeS2-Sb2S3)-E (LUMOFe(III)-6H2O)|; ΔE4 = |E (HOMOFe(III)-6H2O)-E (LUMOFeS2-Sb2S3)|.ΔE1 = |E (HOMOFeS2-Sb2S3)-E (LUMOglucose)|; ΔE2 = |E (HOMOglucose)-E (LUMOFeS2-Sb2S3)|; ΔE3 = |E (HOMOFeS2-Sb2S3)-E (LUMOFe(III)-6H2O)|; ΔE4 = |E (HOMOFe(III)-6H2O)-E (LUMOFeS2-Sb2S3)|.ΔE1 = |E (HOMOFeS2-Sb2S3)-E (LUMOglucose)|; ΔE2 = |E (HOMOglucose)-E (LUMOFeS2-Sb2S3)|; ΔE3 = |E (HOMOFeS2-Sb2S3)-E (LUMOFe(III)-6H2O)|; ΔE4 = |E (HOMOFe(III)-6H2O)-E (LUMOFeS2-Sb2S3)|.ΔE1 = |E (HOMOFeS2-Sb2S3)-E (LUMOglucose)|; ΔE2 = |E (HOMOglucose)-E (LUMOFeS2-Sb2S3)|; ΔE3 = |E (HOMOFeS2-Sb2S3)-E (LUMOFe(III)-6H2O)|; ΔE4 = |E (HOMOFe(III)-6H2O)-E (LUMOFeS2-Sb2S3)|.ΔE1 = |E (HOMOFeS2-Sb2S3)-E (LUMOglucose)|; ΔE2 = |E (HOMOglucose)-E (LUMOFeS2-Sb2S3)|; ΔE3 = |E (HOMOFeS2-Sb2S3)-E (LUMOFe(III)-6H2O)|; ΔE4 = |E (HOMOFe(III)-6H2O)-E (LUMOFeS2-Sb2S3)|._ΔE1 = |E (HOMOFeS2-Sb2S3)-E (LUMOglucose)|; ΔE2 = |E (HOMOglucose)-E (LUMOFeS2-Sb2S3)|; ΔE3 = |E (HOMOFeS2-Sb2S3)-E (LUMOFe(III)-6H2O)|; ΔE4 = |E (HOMOFe(III)-6H2O)-E (LUMOFeS2-Sb2S3)|.ΔE1 = |E (HOMOFeS2-Sb2S3)-E (LUMOglucose)|; ΔE2 = |E (HOMOglucose)-E (LUMOFeS2-Sb2S3)|; ΔE3 = |E (HOMOFeS2-Sb2S3)-E (LUMOFe(III)-6H2O)|; ΔE4 = |E (HOMOFe(III)-6H2O)-E (LUMOFeS2-Sb2S3)|.ΔE1 = |E (HOMOFeS2-Sb2S3)-E (LUMOglucose)|; ΔE2 = |E (HOMOglucose)-E (LUMOFeS2-Sb2S3)|; ΔE3 = |E (HOMOFeS2-Sb2S3)-E (LUMOFe(III)-6H2O)|; ΔE4 = |E (HOMOFe(III)-6H2O)-E (LUMOFeS2-Sb2S3)|.ΔE1 = |E (HOMOFeS2-Sb2S3)-E (LUMOglucose)|; ΔE2 = |E (HOMOglucose)-E (LUMOFeS2-Sb2S3)|; ΔE3 = |E (HOMOFeS2-Sb2S3)-E (LUMOFe(III)-6H2O)|; ΔE4 = |E (HOMOFe(III)-6H2O)-E (LUMOFeS2-Sb2S3)|.ΔE1 = |E (HOMOFeS2-Sb2S3)-E (LUMOglucose)|; ΔE2 = |E (HOMOglucose)-E (LUMOFeS2-Sb2S3)|; ΔE3 = |E (HOMOFeS2-Sb2S3)-E (LUMOFe(III)-6H2O)|; ΔE4 = |E (HOMOFe(III)-6H2O)-E (LUMOFeS2-Sb2S3)|.ΔE1 = |E (HOMOFeS2-Sb2S3)-E (LUMOglucose)|; ΔE2 = |E (HOMOglucose)-E (LUMOFeS2-Sb2S3)|; ΔE3 = |E (HOMOFeS2-Sb2S3)-E (LUMOFe(III)-6H2O)|; ΔE4 = |E (HOMOFe(III)-6H2O)-E (LUMOFeS2-Sb2S3)|.ΔE1 = |E (HOMOFeS2-Sb2S3)-E (LUMOglucose)|; ΔE2 = |E (HOMOglucose)-E (LUMOFeS2-Sb2S3)|; ΔE3 = |E (HOMOFeS2-Sb2S3)-E (LUMOFe(III)-6H2O)|; ΔE4 = |E (HOMOFe(III)-6H2O)-E (LUMOFeS2-Sb2S3)|.ΔE1 = |E (HOMOFeS2-Sb2S3)-E (LUMOglucose)|; ΔE2 = |E (HOMOglucose)-E (LUMOFeS2-Sb2S3)|; ΔE3 = |E (HOMOFeS2-Sb2S3)-E (LUMOFe(III)-6H2O)|; ΔE4 = |E (HOMOFe(III)-6H2O)-E (LUMOFeS2-Sb2S3)|.ΔE1 = |E (HOMOFeS2-Sb2S3)-E (LUMOglucose)|; ΔE2 = |E (HOMOglucose)-E (LUMOFeS2-Sb2S3)|; ΔE3 = |E (HOMOFeS2-Sb2S3)-E (LUMOFe(III)-6H2O)|; ΔE4 = |E (HOMOFe(III)-6H2O)-E (LUMOFeS2-Sb2S3)|._ΔE1 = |E (HOMOFeS2-Sb2S3)-E (LUMOglucose)|; ΔE2 = |E (HOMOglucose)-E (LUMOFeS2-Sb2S3)|; ΔE3 = |E (HOMOFeS2-Sb2S3)-E (LUMOFe(III)-6H2O)|; ΔE4 = |E (HOMOFe(III)-6H2O)-E (LUMOFeS2-Sb2S3)|.ΔE1 = |E (HOMOFeS2-Sb2S3)-E (LUMOglucose)|; ΔE2 = |E (HOMOglucose)-E (LUMOFeS2-Sb2S3)|; ΔE3 = |E (HOMOFeS2-Sb2S3)-E (LUMOFe(III)-6H2O)|; ΔE4 = |E (HOMOFe(III)-6H2O)-E (LUMOFeS2-Sb2S3)|.ΔE1 = |E (HOMOFeS2-Sb2S3)-E (LUMOglucose)|; ΔE2 = |E (HOMOglucose)-E (LUMOFeS2-Sb2S3)|; ΔE3 = |E (HOMOFeS2-Sb2S3)-E (LUMOFe(III)-6H2O)|; ΔE4 = |E (HOMOFe(III)-6H2O)-E (LUMOFeS2-Sb2S3)|.ΔE1 = |E (HOMOFeS2-Sb2S3)-E (LUMOglucose)|; ΔE2 = |E (HOMOglucose)-E (LUMOFeS2-Sb2S3)|; ΔE3 = |E (HOMOFeS2-Sb2S3)-E (LUMOFe(III)-6H2O)|; ΔE4 = |E (HOMOFe(III)-6H2O)-E (LUMOFeS2-Sb2S3)|.ΔE1 = |E (HOMOFeS2-Sb2S3)-E (LUMOglucose)|; ΔE2 = |E (HOMOglucose)-E (LUMOFeS2-Sb2S3)|; ΔE3 = |E (HOMOFeS2-Sb2S3)-E (LUMOFe(III)-6H2O)|; ΔE4 = |E (HOMOFe(III)-6H2O)-E (LUMOFeS2-Sb2S3)|.ΔE1 = |E (HOMOFeS2-Sb2S3)-E (LUMOglucose)|; ΔE2 = |E (HOMOglucose)-E (LUMOFeS2-Sb2S3)|; ΔE3 = |E (HOMOFeS2-Sb2S3)-E (LUMOFe(III)-6H2O)|; ΔE4 = |E (HOMOFe(III)-6H2O)-E (LUMOFeS2-Sb2S3)|.ΔE1 = |E (HOMOFeS2-Sb2S3)-E (LUMOglucose)|; ΔE2 = |E (HOMOglucose)-E (LUMOFeS2-Sb2S3)|; ΔE3 = |E (HOMOFeS2-Sb2S3)-E (LUMOFe(III)-6H2O)|; ΔE4 = |E (HOMOFe(III)-6H2O)-E (LUMOFeS2-Sb2S3)|.ΔE1 = |E (HOMOFeS2-Sb2S3)-E (LUMOglucose)|; ΔE2 = |E (HOMOglucose)-E (LUMOFeS2-Sb2S3)|; ΔE3 = |E (HOMOFeS2-Sb2S3)-E (LUMOFe(III)-6H2O)|; ΔE4 = |E (HOMOFe(III)-6H2O)-E (LUMOFeS2-Sb2S3)|.ΔE1 = |E (HOMOFeS2-Sb2S3)-E (LUMOglucose)|; ΔE2 = |E (HOMOglucose)-E (LUMOFeS2-Sb2S3)|; ΔE3 = |E (HOMOFeS2-Sb2S3)-E (LUMOFe(III)-6H2O)|; ΔE4 = |E (HOMOFe(III)-6H2O)-E (LUMOFeS2-Sb2S3)|.ΔE1 = |E (HOMOFeS2-Sb2S3)-E (LUMOglucose)|; ΔE2 = |E (HOMOglucose)-E (LUMOFeS2-Sb2S3)|; ΔE3 = |E (HOMOFeS2-Sb2S3)-E (LUMOFe(III)-6H2O)|; ΔE4 = |E (HOMOFe(III)-6H2O)-E (LUMOFeS2-Sb2S3)|._ΔE1 = |E (HOMOFeS2-Sb2S3)-E (LUMOglucose)|; ΔE2 = |E (HOMOglucose)-E (LUMOFeS2-Sb2S3)|; ΔE3 = |E (HOMOFeS2-Sb2S3)-E (LUMOFe(III)-6H2O)|; ΔE4 = |E (HOMOFe(III)-6H2O)-E (LUMOFeS2-Sb2S3)|.ΔE1 = |E (HOMOFeS2-Sb2S3)-E (LUMOglucose)|; ΔE2 = |E (HOMOglucose)-E (LUMOFeS2-Sb2S3)|; ΔE3 = |E (HOMOFeS2-Sb2S3)-E (LUMOFe(III)-6H2O)|; ΔE4 = |E (HOMOFe(III)-6H2O)-E (LUMOFeS2-Sb2S3)|.ΔE1 = |E (HOMOFeS2-Sb2S3)-E (LUMOglucose)|; ΔE2 = |E (HOMOglucose)-E (LUMOFeS2-Sb2S3)|; ΔE3 = |E (HOMOFeS2-Sb2S3)-E (LUMOFe(III)-6H2O)|; ΔE4 = |E (HOMOFe(III)-6H2O)-E (LUMOFeS2-Sb2S3)|.ΔE1 = |E (HOMOFeS2-Sb2S3)-E (LUMOglucose)|; ΔE2 = |E (HOMOglucose)-E (LUMOFeS2-Sb2S3)|; ΔE3 = |E (HOMOFeS2-Sb2S3)-E (LUMOFe(III)-6H2O)|; ΔE4 = |E (HOMOFe(III)-6H2O)-E (LUMOFeS2-Sb2S3)|.ΔE1 = |E (HOMOFeS2-Sb2S3)-E (LUMOglucose)|; ΔE2 = |E (HOMOglucose)-E (LUMOFeS2-Sb2S3)|; ΔE3 = |E (HOMOFeS2-Sb2S3)-E (LUMOFe(III)-6H2O)|; ΔE4 = |E (HOMOFe(III)-6H2O)-E (LUMOFeS2-Sb2S3)|.ΔE1 = |E (HOMOFeS2-Sb2S3)-E (LUMOglucose)|; ΔE2 = |E (HOMOglucose)-E (LUMOFeS2-Sb2S3)|; ΔE3 = |E (HOMOFeS2-Sb2S3)-E (LUMOFe(III)-6H2O)|; ΔE4 = |E (HOMOFe(III)-6H2O)-E (LUMOFeS2-Sb2S3)|.ΔE1 = |E (HOMOFeS2-Sb2S3)-E (LUMOglucose)|; ΔE2 = |E (HOMOglucose)-E (LUMOFeS2-Sb2S3)|; ΔE3 = |E (HOMOFeS2-Sb2S3)-E (LUMOFe(III)-6H2O)|; ΔE4 = |E (HOMOFe(III)-6H2O)-E (LUMOFeS2-Sb2S3)|.ΔE1 = |E (HOMOFeS2-Sb2S3)-E (LUMOglucose)|; ΔE2 = |E (HOMOglucose)-E (LUMOFeS2-Sb2S3)|; ΔE3 = |E (HOMOFeS2-Sb2S3)-E (LUMOFe(III)-6H2O)|; ΔE4 = |E (HOMOFe(III)-6H2O)-E (LUMOFeS2-Sb2S3)|.ΔE1 = |E (HOMOFeS2-Sb2S3)-E (LUMOglucose)|; ΔE2 = |E (HOMOglucose)-E (LUMOFeS2-Sb2S3)|; ΔE3 = |E (HOMOFeS2-Sb2S3)-E (LUMOFe(III)-6H2O)|; ΔE4 = |E (HOMOFe(III)-6H2O)-E (LUMOFeS2-Sb2S3)|.ΔE1 = |E (HOMOFeS2-Sb2S3)-E (LUMOglucose)|; ΔE2 = |E (HOMOglucose)-E (LUMOFeS2-Sb2S3)|; ΔE3 = |E (HOMOFeS2-Sb2S3)-E (LUMOFe(III)-6H2O)|; ΔE4 = |E (HOMOFe(III)-6H2O)-E (LUMOFeS2-Sb2S3)|.ΔE1 = |E (HOMOFeS2-Sb2S3)-E (LUMOglucose)|; ΔE2 = |E (HOMOglucose)-E (LUMOFeS2-Sb2S3)|; ΔE3 = |E (HOMOFeS2-Sb2S3)-E (LUMOFe(III)-6H2O)|; ΔE4 = |E (HOMOFe(III)-6H2O)-E (LUMOFeS2-Sb2S3)|._ΔE1 = |E (HOMOFeS2-Sb2S3)-E (LUMOglucose)|; ΔE2 = |E (HOMOglucose)-E (LUMOFeS2-Sb2S3)|; ΔE3 = |E (HOMOFeS2-Sb2S3)-E (LUMOFe(III)-6H2O)|; ΔE4 = |E (HOMOFe(III)-6H2O)-E (LUMOFeS2-Sb2S3)|.ΔE1 = |E (HOMOFeS2-Sb2S3)-E (LUMOglucose)|; ΔE2 = |E (HOMOglucose)-E (LUMOFeS2-Sb2S3)|; ΔE3 = |E (HOMOFeS2-Sb2S3)-E (LUMOFe(III)-6H2O)|; ΔE4 = |E (HOMOFe(III)-6H2O)-E (LUMOFeS2-Sb2S3)|.ΔE1 = |E (HOMOFeS2-Sb2S3)-E (LUMOglucose)|; ΔE2 = |E (HOMOglucose)-E (LUMOFeS2-Sb2S3)|; ΔE3 = |E (HOMOFeS2-Sb2S3)-E (LUMOFe(III)-6H2O)|; ΔE4 = |E (HOMOFe(III)-6H2O)-E (LUMOFeS2-Sb2S3)|.ΔE1 = |E (HOMOFeS2-Sb2S3)-E (LUMOglucose)|; ΔE2 = |E (HOMOglucose)-E (LUMOFeS2-Sb2S3)|; ΔE3 = |E (HOMOFeS2-Sb2S3)-E (LUMOFe(III)-6H2O)|; ΔE4 = |E (HOMOFe(III)-6H2O)-E (LUMOFeS2-Sb2S3)|.ΔE1 = |E (HOMOFeS2-Sb2S3)-E (LUMOglucose)|; ΔE2 = |E (HOMOglucose)-E (LUMOFeS2-Sb2S3)|; ΔE3 = |E (HOMOFeS2-Sb2S3)-E (LUMOFe(III)-6H2O)|; ΔE4 = |E (HOMOFe(III)-6H2O)-E (LUMOFeS2-Sb2S3)|.ΔE1 = |E (HOMOFeS2-Sb2S3)-E (LUMOglucose)|; ΔE2 = |E (HOMOglucose)-E (LUMOFeS2-Sb2S3)|; ΔE3 = |E (HOMOFeS2-Sb2S3)-E (LUMOFe(III)-6H2O)|; ΔE4 = |E (HOMOFe(III)-6H2O)-E (LUMOFeS2-Sb2S3)|.ΔE1 = |E (HOMOFeS2-Sb2S3)-E (LUMOglucose)|; ΔE2 = |E (HOMOglucose)-E (LUMOFeS2-Sb2S3)|; ΔE3 = |E (HOMOFeS2-Sb2S3)-E (LUMOFe(III)-6H2O)|; ΔE4 = |E (HOMOFe(III)-6H2O)-E (LUMOFeS2-Sb2S3)|.ΔE1 = |E (HOMOFeS2-Sb2S3)-E (LUMOglucose)|; ΔE2 = |E (HOMOglucose)-E (LUMOFeS2-Sb2S3)|; ΔE3 = |E (HOMOFeS2-Sb2S3)-E (LUMOFe(III)-6H2O)|; ΔE4 = |E (HOMOFe(III)-6H2O)-E (LUMOFeS2-Sb2S3)|.ΔE1 = |E (HOMOFeS2-Sb2S3)-E (LUMOglucose)|; ΔE2 = |E (HOMOglucose)-E (LUMOFeS2-Sb2S3)|; ΔE3 = |E (HOMOFeS2-Sb2S3)-E (LUMOFe(III)-6H2O)|; ΔE4 = |E (HOMOFe(III)-6H2O)-E (LUMOFeS2-Sb2S3)|.ΔE1 = |E (HOMOFeS2-Sb2S3)-E (LUMOglucose)|; ΔE2 = |E (HOMOglucose)-E (LUMOFeS2-Sb2S3)|; ΔE3 = |E (HOMOFeS2-Sb2S3)-E (LUMOFe(III)-6H2O)|; ΔE4 = |E (HOMOFe(III)-6H2O)-E (LUMOFeS2-Sb2S3)|._ΔE1 = |E (HOMOFeS2-Sb2S3)-E (LUMOglucose)|; ΔE2 = |E (HOMOglucose)-E (LUMOFeS2-Sb2S3)|; ΔE3 = |E (HOMOFeS2-Sb2S3)-E (LUMOFe(III)-6H2O)|; ΔE4 = |E (HOMOFe(III)-6H2O)-E (LUMOFeS2-Sb2S3)|.ΔE1 = |E (HOMOFeS2-Sb2S3)-E (LUMOglucose)|; ΔE2 = |E (HOMOglucose)-E (LUMOFeS2-Sb2S3)|; ΔE3 = |E (HOMOFeS2-Sb2S3)-E (LUMOFe(III)-6H2O)|; ΔE4 = |E (HOMOFe(III)-6H2O)-E (LUMOFeS2-Sb2S3)|.ΔE1 = |E (HOMOFeS2-Sb2S3)-E (LUMOglucose)|; ΔE2 = |E (HOMOglucose)-E (LUMOFeS2-Sb2S3)|; ΔE3 = |E (HOMOFeS2-Sb2S3)-E (LUMOFe(III)-6H2O)|; ΔE4 = |E (HOMOFe(III)-6H2O)-E (LUMOFeS2-Sb2S3)|.ΔE1 = |E (HOMOFeS2-Sb2S3)-E (LUMOglucose)|; ΔE2 = |E (HOMOglucose)-E (LUMOFeS2-Sb2S3)|; ΔE3 = |E (HOMOFeS2-Sb2S3)-E (LUMOFe(III)-6H2O)|; ΔE4 = |E (HOMOFe(III)-6H2O)-E (LUMOFeS2-Sb2S3)|.ΔE1 = |E (HOMOFeS2-Sb2S3)-E (LUMOglucose)|; ΔE2 = |E (HOMOglucose)-E (LUMOFeS2-Sb2S3)|; ΔE3 = |E (HOMOFeS2-Sb2S3)-E (LUMOFe(III)-6H2O)|; ΔE4 = |E (HOMOFe(III)-6H2O)-E (LUMOFeS2-Sb2S3)|.ΔE1 = |E (HOMOFeS2-Sb2S3)-E (LUMOglucose)|; ΔE2 = |E (HOMOglucose)-E (LUMOFeS2-Sb2S3)|; ΔE3 = |E (HOMOFeS2-Sb2S3)-E (LUMOFe(III)-6H2O)|; ΔE4 = |E (HOMOFe(III)-6H2O)-E (LUMOFeS2-Sb2S3)|.ΔE1 = |E (HOMOFeS2-Sb2S3)-E (LUMOglucose)|; ΔE2 = |E (HOMOglucose)-E (LUMOFeS2-Sb2S3)|; ΔE3 = |E (HOMOFeS2-Sb2S3)-E (LUMOFe(III)-6H2O)|; ΔE4 = |E (HOMOFe(III)-6H2O)-E (LUMOFeS2-Sb2S3)|.ΔE1 = |E (HOMOFeS2-Sb2S3)-E (LUMOglucose)|; ΔE2 = |E (HOMOglucose)-E (LUMOFeS2-Sb2S3)|; ΔE3 = |E (HOMOFeS2-Sb2S3)-E (LUMOFe(III)-6H2O)|; ΔE4 = |E (HOMOFe(III)-6H2O)-E (LUMOFeS2-Sb2S3)|.ΔE1 = |E (HOMOFeS2-Sb2S3)-E (LUMOglucose)|; ΔE2 = |E (HOMOglucose)-E (LUMOFeS2-Sb2S3)|; ΔE3 = |E (HOMOFeS2-Sb2S3)-E (LUMOFe(III)-6H2O)|; ΔE4 = |E (HOMOFe(III)-6H2O)-E (LUMOFeS2-Sb2S3)|._ΔE1 = |E (HOMOFeS2-Sb2S3)-E (LUMOglucose)|; ΔE2 = |E (HOMOglucose)-E (LUMOFeS2-Sb2S3)|; ΔE3 = |E (HOMOFeS2-Sb2S3)-E (LUMOFe(III)-6H2O)|; ΔE4 = |E (HOMOFe(III)-6H2O)-E (LUMOFeS2-Sb2S3)|.ΔE1 = |E (HOMOFeS2-Sb2S3)-E (LUMOglucose)|; ΔE2 = |E (HOMOglucose)-E (LUMOFeS2-Sb2S3)|; ΔE3 = |E (HOMOFeS2-Sb2S3)-E (LUMOFe(III)-6H2O)|; ΔE4 = |E (HOMOFe(III)-6H2O)-E (LUMOFeS2-Sb2S3)|.ΔE1 = |E (HOMOFeS2-Sb2S3)-E (LUMOglucose)|; ΔE2 = |E (HOMOglucose)-E (LUMOFeS2-Sb2S3)|; ΔE3 = |E (HOMOFeS2-Sb2S3)-E (LUMOFe(III)-6H2O)|; ΔE4 = |E (HOMOFe(III)-6H2O)-E (LUMOFeS2-Sb2S3)|.ΔE1 = |E (HOMOFeS2-Sb2S3)-E (LUMOglucose)|; ΔE2 = |E (HOMOglucose)-E (LUMOFeS2-Sb2S3)|; ΔE3 = |E (HOMOFeS2-Sb2S3)-E (LUMOFe(III)-6H2O)|; ΔE4 = |E (HOMOFe(III)-6H2O)-E (LUMOFeS2-Sb2S3)|.ΔE1 = |E (HOMOFeS2-Sb2S3)-E (LUMOglucose)|; ΔE2 = |E (HOMOglucose)-E (LUMOFeS2-Sb2S3)|; ΔE3 = |E (HOMOFeS2-Sb2S3)-E (LUMOFe(III)-6H2O)|; ΔE4 = |E (HOMOFe(III)-6H2O)-E (LUMOFeS2-Sb2S3)|.ΔE1 = |E (HOMOFeS2-Sb2S3)-E (LUMOglucose)|; ΔE2 = |E (HOMOglucose)-E (LUMOFeS2-Sb2S3)|; ΔE3 = |E (HOMOFeS2-Sb2S3)-E (LUMOFe(III)-6H2O)|; ΔE4 = |E (HOMOFe(III)-6H2O)-E (LUMOFeS2-Sb2S3)|.ΔE1 = |E (HOMOFeS2-Sb2S3)-E (LUMOglucose)|; ΔE2 = |E (HOMOglucose)-E (LUMOFeS2-Sb2S3)|; ΔE3 = |E (HOMOFeS2-Sb2S3)-E (LUMOFe(III)-6H2O)|; ΔE4 = |E (HOMOFe(III)-6H2O)-E (LUMOFeS2-Sb2S3)|.ΔE1 = |E (HOMOFeS2-Sb2S3)-E (LUMOglucose)|; ΔE2 = |E (HOMOglucose)-E (LUMOFeS2-Sb2S3)|; ΔE3 = |E (HOMOFeS2-Sb2S3)-E (LUMOFe(III)-6H2O)|; ΔE4 = |E (HOMOFe(III)-6H2O)-E (LUMOFeS2-Sb2S3)|.ΔE1 = |E (HOMOFeS2-Sb2S3)-E (LUMOglucose)|; ΔE2 = |E (HOMOglucose)-E (LUMOFeS2-Sb2S3)|; ΔE3 = |E (HOMOFeS2-Sb2S3)-E (LUMOFe(III)-6H2O)|; ΔE4 = |E (HOMOFe(III)-6H2O)-E (LUMOFeS2-Sb2S3)|.ΔE1 = |E (HOMOFeS2-Sb2S3)-E (LUMOglucose)|; ΔE2 = |E (HOMOglucose)-E (LUMOFeS2-Sb2S3)|; ΔE3 = |E (HOMOFeS2-Sb2S3)-E (LUMOFe(III)-6H2O)|; ΔE4 = |E (HOMOFe(III)-6H2O)-E (LUMOFeS2-Sb2S3)|.ΔE1 = |E (HOMOFeS2-Sb2S3)-E (LUMOglucose)|; ΔE2 = |E (HOMOglucose)-E (LUMOFeS2-Sb2S3)|; ΔE3 = |E (HOMOFeS2-Sb2S3)-E (LUMOFe(III)-6H2O)|; ΔE4 = |E (HOMOFe(III)-6H2O)-E (LUMOFeS2-Sb2S3)|.ΔE1 = |E (HOMOFeS2-Sb2S3)-E (LUMOglucose)|; ΔE2 = |E (HOMOglucose)-E (LUMOFeS2-Sb2S3)|; ΔE3 = |E (HOMOFeS2-Sb2S3)-E (LUMOFe(III)-6H2O)|; ΔE4 = |E (HOMOFe(III)-6H2O)-E (LUMOFeS2-Sb2S3)|._ΔE1 = |E (HOMOFeS2-Sb2S3)-E (LUMOglucose)|; ΔE2 = |E (HOMOglucose)-E (LUMOFeS2-Sb2S3)|; ΔE3 = |E (HOMOFeS2-Sb2S3)-E (LUMOFe(III)-6H2O)|; ΔE4 = |E (HOMOFe(III)-6H2O)-E (LUMOFeS2-Sb2S3)|.ΔE1 = |E (HOMOFeS2-Sb2S3)-E (LUMOglucose)|; ΔE2 = |E (HOMOglucose)-E (LUMOFeS2-Sb2S3)|; ΔE3 = |E (HOMOFeS2-Sb2S3)-E (LUMOFe(III)-6H2O)|; ΔE4 = |E (HOMOFe(III)-6H2O)-E (LUMOFeS2-Sb2S3)|.ΔE1 = |E (HOMOFeS2-Sb2S3)-E (LUMOglucose)|; ΔE2 = |E (HOMOglucose)-E (LUMOFeS2-Sb2S3)|; ΔE3 = |E (HOMOFeS2-Sb2S3)-E (LUMOFe(III)-6H2O)|; ΔE4 = |E (HOMOFe(III)-6H2O)-E (LUMOFeS2-Sb2S3)|.ΔE1 = |E (HOMOFeS2-Sb2S3)-E (LUMOglucose)|; ΔE2 = |E (HOMOglucose)-E (LUMOFeS2-Sb2S3)|; ΔE3 = |E (HOMOFeS2-Sb2S3)-E (LUMOFe(III)-6H2O)|; ΔE4 = |E (HOMOFe(III)-6H2O)-E (LUMOFeS2-Sb2S3)|.ΔE1 = |E (HOMOFeS2-Sb2S3)-E (LUMOglucose)|; ΔE2 = |E (HOMOglucose)-E (LUMOFeS2-Sb2S3)|; ΔE3 = |E (HOMOFeS2-Sb2S3)-E (LUMOFe(III)-6H2O)|; ΔE4 = |E (HOMOFe(III)-6H2O)-E (LUMOFeS2-Sb2S3)|.ΔE1 = |E (HOMOFeS2-Sb2S3)-E (LUMOglucose)|; ΔE2 = |E (HOMOglucose)-E (LUMOFeS2-Sb2S3)|; ΔE3 = |E (HOMOFeS2-Sb2S3)-E (LUMOFe(III)-6H2O)|; ΔE4 = |E (HOMOFe(III)-6H2O)-E (LUMOFeS2-Sb2S3)|.ΔE1 = |E (HOMOFeS2-Sb2S3)-E (LUMOglucose)|; ΔE2 = |E (HOMOglucose)-E (LUMOFeS2-Sb2S3)|; ΔE3 = |E (HOMOFeS2-Sb2S3)-E (LUMOFe(III)-6H2O)|; ΔE4 = |E (HOMOFe(III)-6H2O)-E (LUMOFeS2-Sb2S3)|.ΔE1 = |E (HOMOFeS2-Sb2S3)-E (LUMOglucose)|; ΔE2 = |E (HOMOglucose)-E (LUMOFeS2-Sb2S3)|; ΔE3 = |E (HOMOFeS2-Sb2S3)-E (LUMOFe(III)-6H2O)|; ΔE4 = |E (HOMOFe(III)-6H2O)-E (LUMOFeS2-Sb2S3)|.ΔE1 = |E (HOMOFeS2-Sb2S3)-E (LUMOglucose)|; ΔE2 = |E (HOMOglucose)-E (LUMOFeS2-Sb2S3)|; ΔE3 = |E (HOMOFeS2-Sb2S3)-E (LUMOFe(III)-6H2O)|; ΔE4 = |E (HOMOFe(III)-6H2O)-E (LUMOFeS2-Sb2S3)|.ΔE1 = |E (HOMOFeS2-Sb2S3)-E (LUMOglucose)|; ΔE2 = |E (HOMOglucose)-E (LUMOFeS2-Sb2S3)|; ΔE3 = |E (HOMOFeS2-Sb2S3)-E (LUMOFe(III)-6H2O)|; ΔE4 = |E (HOMOFe(III)-6H2O)-E (LUMOFeS2-Sb2S3)|.ΔE1 = |E (HOMOFeS2-Sb2S3)-E (LUMOglucose)|; ΔE2 = |E (HOMOglucose)-E (LUMOFeS2-Sb2S3)|; ΔE3 = |E (HOMOFeS2-Sb2S3)-E (LUMOFe(III)-6H2O)|; ΔE4 = |E (HOMOFe(III)-6H2O)-E (LUMOFeS2-Sb2S3)|._ΔE1 = |E (HOMOFeS2-Sb2S3)-E (LUMOglucose)|; ΔE2 = |E (HOMOglucose)-E (LUMOFeS2-Sb2S3)|; ΔE3 = |E (HOMOFeS2-Sb2S3)-E (LUMOFe(III)-6H2O)|; ΔE4 = |E (HOMOFe(III)-6H2O)-E (LUMOFeS2-Sb2S3)|.ΔE1 = |E (HOMOFeS2-Sb2S3)-E (LUMOglucose)|; ΔE2 = |E (HOMOglucose)-E (LUMOFeS2-Sb2S3)|; ΔE3 = |E (HOMOFeS2-Sb2S3)-E (LUMOFe(III)-6H2O)|; ΔE4 = |E (HOMOFe(III)-6H2O)-E (LUMOFeS2-Sb2S3)|.ΔE1 = |E (HOMOFeS2-Sb2S3)-E (LUMOglucose)|; ΔE2 = |E (HOMOglucose)-E (LUMOFeS2-Sb2S3)|; ΔE3 = |E (HOMOFeS2-Sb2S3)-E (LUMOFe(III)-6H2O)|; ΔE4 = |E (HOMOFe(III)-6H2O)-E (LUMOFeS2-Sb2S3)|.ΔE1 = |E (HOMOFeS2-Sb2S3)-E (LUMOglucose)|; ΔE2 = |E (HOMOglucose)-E (LUMOFeS2-Sb2S3)|; ΔE3 = |E (HOMOFeS2-Sb2S3)-E (LUMOFe(III)-6H2O)|; ΔE4 = |E (HOMOFe(III)-6H2O)-E (LUMOFeS2-Sb2S3)|.ΔE1 = |E (HOMOFeS2-Sb2S3)-E (LUMOglucose)|; ΔE2 = |E (HOMOglucose)-E (LUMOFeS2-Sb2S3)|; ΔE3 = |E (HOMOFeS2-Sb2S3)-E (LUMOFe(III)-6H2O)|; ΔE4 = |E (HOMOFe(III)-6H2O)-E (LUMOFeS2-Sb2S3)|.ΔE1 = |E (HOMOFeS2-Sb2S3)-E (LUMOglucose)|; ΔE2 = |E (HOMOglucose)-E (LUMOFeS2-Sb2S3)|; ΔE3 = |E (HOMOFeS2-Sb2S3)-E (LUMOFe(III)-6H2O)|; ΔE4 = |E (HOMOFe(III)-6H2O)-E (LUMOFeS2-Sb2S3)|.ΔE1 = |E (HOMOFeS2-Sb2S3)-E (LUMOglucose)|; ΔE2 = |E (HOMOglucose)-E (LUMOFeS2-Sb2S3)|; ΔE3 = |E (HOMOFeS2-Sb2S3)-E (LUMOFe(III)-6H2O)|; ΔE4 = |E (HOMOFe(III)-6H2O)-E (LUMOFeS2-Sb2S3)|.ΔE1 = |E (HOMOFeS2-Sb2S3)-E (LUMOglucose)|; ΔE2 = |E (HOMOglucose)-E (LUMOFeS2-Sb2S3)|; ΔE3 = |E (HOMOFeS2-Sb2S3)-E (LUMOFe(III)-6H2O)|; ΔE4 = |E (HOMOFe(III)-6H2O)-E (LUMOFeS2-Sb2S3)|.ΔE1 = |E (HOMOFeS2-Sb2S3)-E (LUMOglucose)|; ΔE2 = |E (HOMOglucose)-E (LUMOFeS2-Sb2S3)|; ΔE3 = |E (HOMOFeS2-Sb2S3)-E (LUMOFe(III)-6H2O)|; ΔE4 = |E (HOMOFe(III)-6H2O)-E (LUMOFeS2-Sb2S3)|.ΔE1 = |E (HOMOFeS2-Sb2S3)-E (LUMOglucose)|; ΔE2 = |E (HOMOglucose)-E (LUMOFeS2-Sb2S3)|; ΔE3 = |E (HOMOFeS2-Sb2S3)-E (LUMOFe(III)-6H2O)|; ΔE4 = |E (HOMOFe(III)-6H2O)-E (LUMOFeS2-Sb2S3)|.ΔE1 = |E (HOMOFeS2-Sb2S3)-E (LUMOglucose)|; ΔE2 = |E (HOMOglucose)-E (LUMOFeS2-Sb2S3)|; ΔE3 = |E (HOMOFeS2-Sb2S3)-E (LUMOFe(III)-6H2O)|; ΔE4 = |E (HOMOFe(III)-6H2O)-E (LUMOFeS2-Sb2S3)|.ΔE1 = |E (HOMOFeS2-Sb2S3)-E (LUMOglucose)|; ΔE2 = |E (HOMOglucose)-E (LUMOFeS2-Sb2S3)|; ΔE3 = |E (HOMOFeS2-Sb2S3)-E (LUMOFe(III)-6H2O)|; ΔE4 = |E (HOMOFe(III)-6H2O)-E (LUMOFeS2-Sb2S3)|._ΔE1 = |E (HOMOFeS2-Sb2S3)-E (LUMOglucose)|; ΔE2 = |E (HOMOglucose)-E (LUMOFeS2-Sb2S3)|; ΔE3 = |E (HOMOFeS2-Sb2S3)-E (LUMOFe(III)-6H2O)|; ΔE4 = |E (HOMOFe(III)-6H2O)-E (LUMOFeS2-Sb2S3)|.ΔE1 = |E (HOMOFeS2-Sb2S3)-E (LUMOglucose)|; ΔE2 = |E (HOMOglucose)-E (LUMOFeS2-Sb2S3)|; ΔE3 = |E (HOMOFeS2-Sb2S3)-E (LUMOFe(III)-6H2O)|; ΔE4 = |E (HOMOFe(III)-6H2O)-E (LUMOFeS2-Sb2S3)|.ΔE1 = |E (HOMOFeS2-Sb2S3)-E (LUMOglucose)|; ΔE2 = |E (HOMOglucose)-E (LUMOFeS2-Sb2S3)|; ΔE3 = |E (HOMOFeS2-Sb2S3)-E (LUMOFe(III)-6H2O)|; ΔE4 = |E (HOMOFe(III)-6H2O)-E (LUMOFeS2-Sb2S3)|.ΔE1 = |E (HOMOFeS2-Sb2S3)-E (LUMOglucose)|; ΔE2 = |E (HOMOglucose)-E (LUMOFeS2-Sb2S3)|; ΔE3 = |E (HOMOFeS2-Sb2S3)-E (LUMOFe(III)-6H2O)|; ΔE4 = |E (HOMOFe(III)-6H2O)-E (LUMOFeS2-Sb2S3)|.ΔE1 = |E (HOMOFeS2-Sb2S3)-E (LUMOglucose)|; ΔE2 = |E (HOMOglucose)-E (LUMOFeS2-Sb2S3)|; ΔE3 = |E (HOMOFeS2-Sb2S3)-E (LUMOFe(III)-6H2O)|; ΔE4 = |E (HOMOFe(III)-6H2O)-E (LUMOFeS2-Sb2S3)|.ΔE1 = |E (HOMOFeS2-Sb2S3)-E (LUMOglucose)|; ΔE2 = |E (HOMOglucose)-E (LUMOFeS2-Sb2S3)|; ΔE3 = |E (HOMOFeS2-Sb2S3)-E (LUMOFe(III)-6H2O)|; ΔE4 = |E (HOMOFe(III)-6H2O)-E (LUMOFeS2-Sb2S3)|.ΔE1 = |E (HOMOFeS2-Sb2S3)-E (LUMOglucose)|; ΔE2 = |E (HOMOglucose)-E (LUMOFeS2-Sb2S3)|; ΔE3 = |E (HOMOFeS2-Sb2S3)-E (LUMOFe(III)-6H2O)|; ΔE4 = |E (HOMOFe(III)-6H2O)-E (LUMOFeS2-Sb2S3)|.ΔE1 = |E (HOMOFeS2-Sb2S3)-E (LUMOglucose)|; ΔE2 = |E (HOMOglucose)-E (LUMOFeS2-Sb2S3)|; ΔE3 = |E (HOMOFeS2-Sb2S3)-E (LUMOFe(III)-6H2O)|; ΔE4 = |E (HOMOFe(III)-6H2O)-E (LUMOFeS2-Sb2S3)|.ΔE1 = |E (HOMOFeS2-Sb2S3)-E (LUMOglucose)|; ΔE2 = |E (HOMOglucose)-E (LUMOFeS2-Sb2S3)|; ΔE3 = |E (HOMOFeS2-Sb2S3)-E (LUMOFe(III)-6H2O)|; ΔE4 = |E (HOMOFe(III)-6H2O)-E (LUMOFeS2-Sb2S3)|._ΔE1 = |E (HOMOFeS2-Sb2S3)-E (LUMOglucose)|; ΔE2 = |E (HOMOglucose)-E (LUMOFeS2-Sb2S3)|; ΔE3 = |E (HOMOFeS2-Sb2S3)-E (LUMOFe(III)-6H2O)|; ΔE4 = |E (HOMOFe(III)-6H2O)-E (LUMOFeS2-Sb2S3)|.ΔE1 = |E (HOMOFeS2-Sb2S3)-E (LUMOglucose)|; ΔE2 = |E (HOMOglucose)-E (LUMOFeS2-Sb2S3)|; ΔE3 = |E (HOMOFeS2-Sb2S3)-E (LUMOFe(III)-6H2O)|; ΔE4 = |E (HOMOFe(III)-6H2O)-E (LUMOFeS2-Sb2S3)|.ΔE1 = |E (HOMOFeS2-Sb2S3)-E (LUMOglucose)|; ΔE2 = |E (HOMOglucose)-E (LUMOFeS2-Sb2S3)|; ΔE3 = |E (HOMOFeS2-Sb2S3)-E (LUMOFe(III)-6H2O)|; ΔE4 = |E (HOMOFe(III)-6H2O)-E (LUMOFeS2-Sb2S3)|.ΔE1 = |E (HOMOFeS2-Sb2S3)-E (LUMOglucose)|; ΔE2 = |E (HOMOglucose)-E (LUMOFeS2-Sb2S3)|; ΔE3 = |E (HOMOFeS2-Sb2S3)-E (LUMOFe(III)-6H2O)|; ΔE4 = |E (HOMOFe(III)-6H2O)-E (LUMOFeS2-Sb2S3)|.ΔE1 = |E (HOMOFeS2-Sb2S3)-E (LUMOglucose)|; ΔE2 = |E (HOMOglucose)-E (LUMOFeS2-Sb2S3)|; ΔE3 = |E (HOMOFeS2-Sb2S3)-E (LUMOFe(III)-6H2O)|; ΔE4 = |E (HOMOFe(III)-6H2O)-E (LUMOFeS2-Sb2S3)|.ΔE1 = |E (HOMOFeS2-Sb2S3)-E (LUMOglucose)|; ΔE2 = |E (HOMOglucose)-E (LUMOFeS2-Sb2S3)|; ΔE3 = |E (HOMOFeS2-Sb2S3)-E (LUMOFe(III)-6H2O)|; ΔE4 = |E (HOMOFe(III)-6H2O)-E (LUMOFeS2-Sb2S3)|.ΔE1 = |E (HOMOFeS2-Sb2S3)-E (LUMOglucose)|; ΔE2 = |E (HOMOglucose)-E (LUMOFeS2-Sb2S3)|; ΔE3 = |E (HOMOFeS2-Sb2S3)-E (LUMOFe(III)-6H2O)|; ΔE4 = |E (HOMOFe(III)-6H2O)-E (LUMOFeS2-Sb2S3)|.ΔE1 = |E (HOMOFeS2-Sb2S3)-E (LUMOglucose)|; ΔE2 = |E (HOMOglucose)-E (LUMOFeS2-Sb2S3)|; ΔE3 = |E (HOMOFeS2-Sb2S3)-E (LUMOFe(III)-6H2O)|; ΔE4 = |E (HOMOFe(III)-6H2O)-E (LUMOFeS2-Sb2S3)|.ΔE1 = |E (HOMOFeS2-Sb2S3)-E (LUMOglucose)|; ΔE2 = |E (HOMOglucose)-E (LUMOFeS2-Sb2S3)|; ΔE3 = |E (HOMOFeS2-Sb2S3)-E (LUMOFe(III)-6H2O)|; ΔE4 = |E (HOMOFe(III)-6H2O)-E (LUMOFeS2-Sb2S3)|.ΔE1 = |E (HOMOFeS2-Sb2S3)-E (LUMOglucose)|; ΔE2 = |E (HOMOglucose)-E (LUMOFeS2-Sb2S3)|; ΔE3 = |E (HOMOFeS2-Sb2S3)-E (LUMOFe(III)-6H2O)|; ΔE4 = |E (HOMOFe(III)-6H2O)-E (LUMOFeS2-Sb2S3)|._ΔE1 = |E (HOMOFeS2-Sb2S3)-E (LUMOglucose)|; ΔE2 = |E (HOMOglucose)-E (LUMOFeS2-Sb2S3)|; ΔE3 = |E (HOMOFeS2-Sb2S3)-E (LUMOFe(III)-6H2O)|; ΔE4 = |E (HOMOFe(III)-6H2O)-E (LUMOFeS2-Sb2S3)|.ΔE1 = |E (HOMOFeS2-Sb2S3)-E (LUMOglucose)|; ΔE2 = |E (HOMOglucose)-E (LUMOFeS2-Sb2S3)|; ΔE3 = |E (HOMOFeS2-Sb2S3)-E (LUMOFe(III)-6H2O)|; ΔE4 = |E (HOMOFe(III)-6H2O)-E (LUMOFeS2-Sb2S3)|.ΔE1 = |E (HOMOFeS2-Sb2S3)-E (LUMOglucose)|; ΔE2 = |E (HOMOglucose)-E (LUMOFeS2-Sb2S3)|; ΔE3 = |E (HOMOFeS2-Sb2S3)-E (LUMOFe(III)-6H2O)|; ΔE4 = |E (HOMOFe(III)-6H2O)-E (LUMOFeS2-Sb2S3)|.

**Figure 12:**
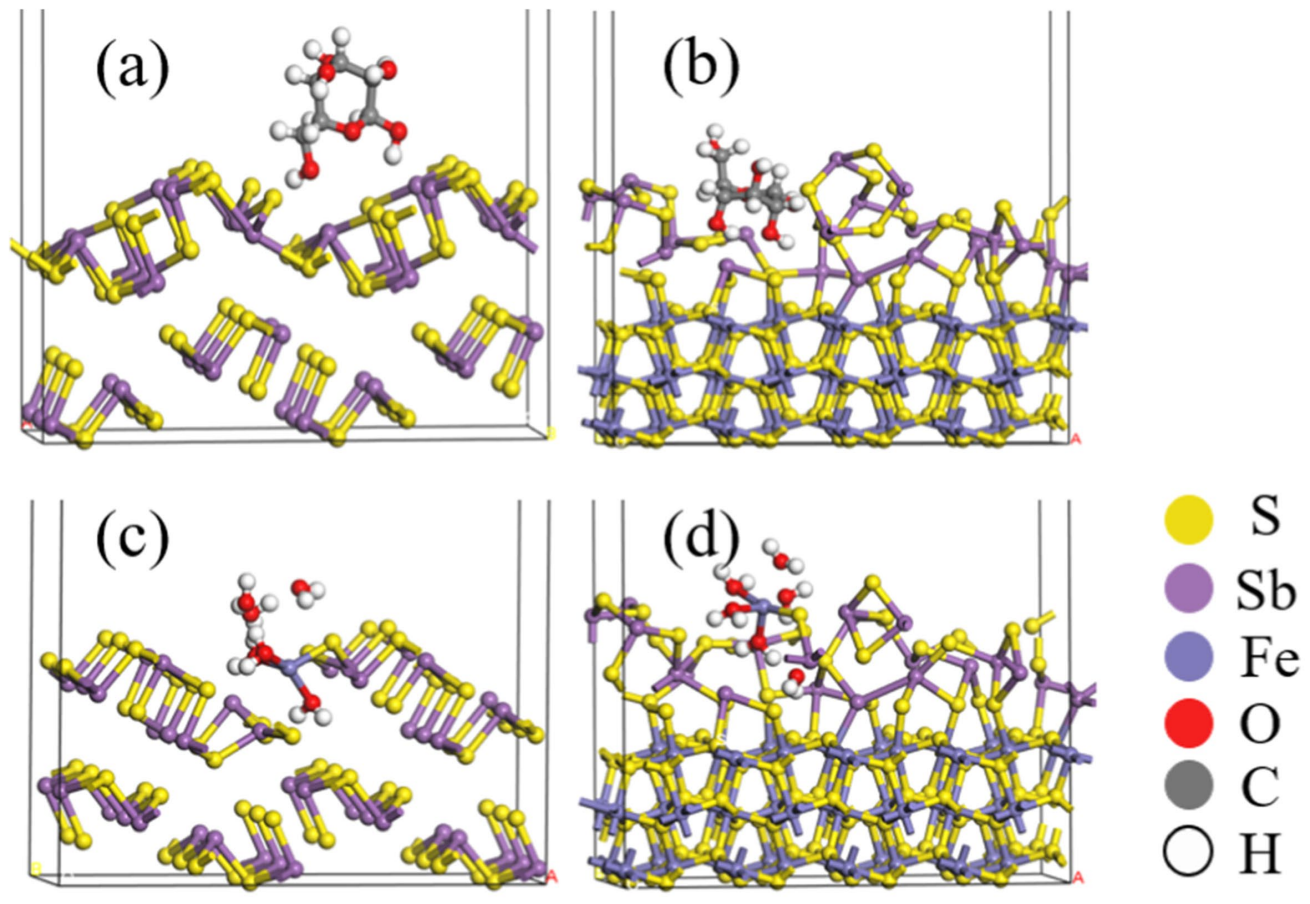
Optimized configurations of glucose **(A,B)** or Fe(III)-6H_2_O **(C,D)** adsorbed on Sb_2_S_3_, FeS_2_-Sb_2_S_3_.

**Figure 13:**
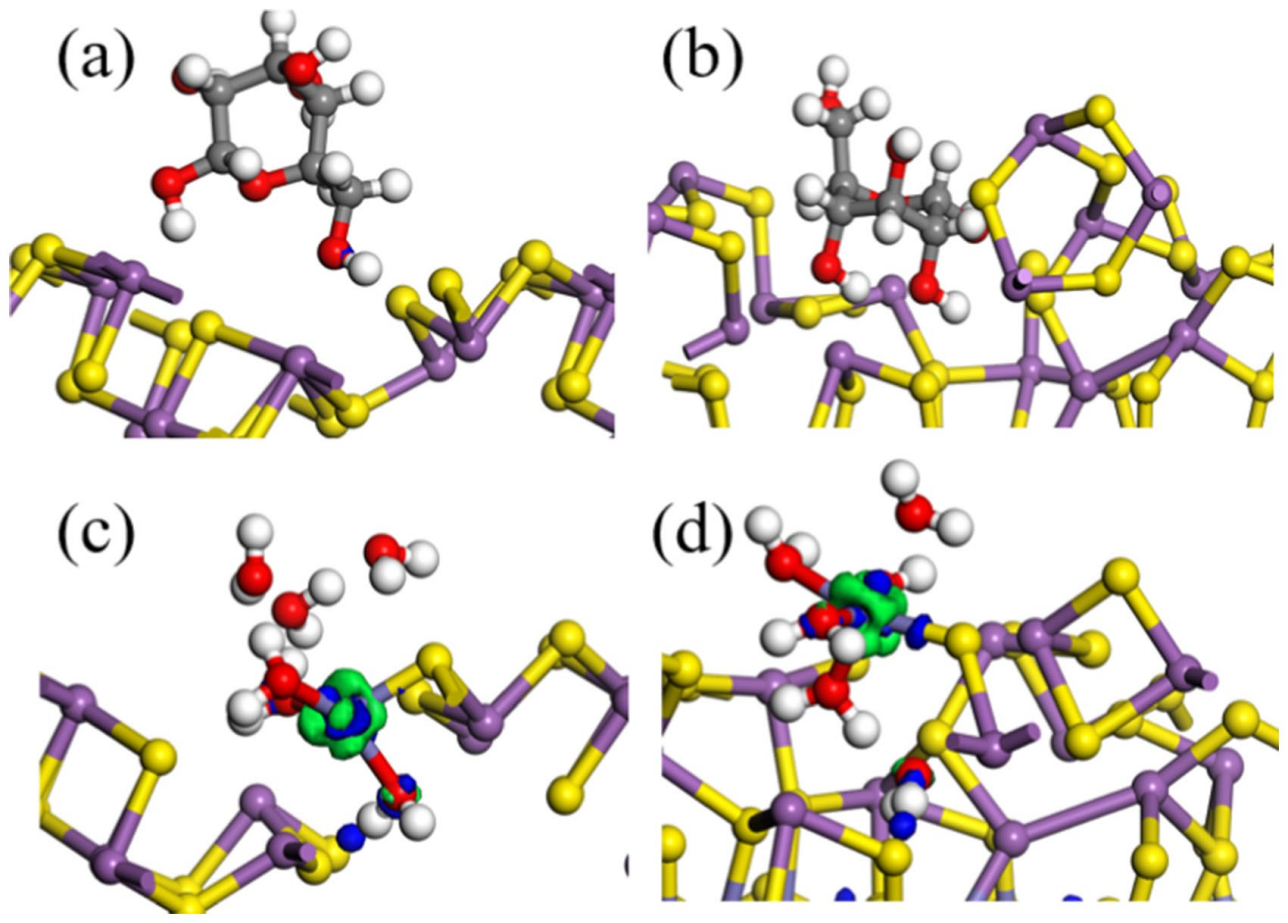
**(A,B)** Electron density difference for the interaction between glucose and the Sb_2_S_3_, FeS_2_-Sb_2_S_3_ surface; **(C,D)** electron density difference for the interaction between Fe(III)-6H_2_O and the Sb_2_S_3_, or FeS_2_-Sb_2_S_3_ surface. The green area represents electron loss, and the blue contours indicate an increase in the electron density.

Figures 12A,B and Table 5 show that after adding FeS_2_, the bond length (Å) between glucose and the Sb_2_S_3_ surface becomes shorter, from 3.093 Å to 2.907 Å, and the adsorption energy decreases from 0.35 eV to 0.16 eV, indicating that FeS_2_ can promote the adsorption of bacteria on the Sb_2_S_3_ surface, thereby enhancing bioleaching. Figures 12C,D and Table 5 show that Fe(III)-6H_2_O can adsorb on the Sb_2_S_3_ surface and has a shorter bond length and lower adsorption energy than glucose. Table 6 shows that after Fe(III)-6H_2_O adsorption, the charge change is more greater (−0.21) than that of glucose (−0.03). The EDD (Electron density difference) result (Figure 13) shows that there is more electron transfer between Fe(III)-6H_2_O and Sb_2_S_3,_ implying stronger adsorption. Notably, the results also show that FeS_2_ can promote the adsorption of Fe(III)-6H_2_O on the Sb_2_S_3_ surface, possibly because adding FeS_2_ makes the Sb_2_S_3_ surface disordered, which is beneficial for Fe(III)-6H_2_O adsorption. In general, trivalent iron derived from FeS_2_ has a stronger oxidation effect on Sb_2_S_3_ during the bioleaching process.

**Table 5:** Mulliken bond population analysis of FeS_2_-Sb_2_S_3_.

	Bond (shorter one)	Bond lengths (Å)	Populations	Adsorption energies (eV)
Glucose-Sb_2_S_3_	O-Sb	3.093	–	0.35
Glucose-FeS_2_-Sb_2_S_3_	O-Sb	2.907	–	0.16
Fe^3+^-6H_2_O- Sb_2_S_3_	Fe-S	2.276	0.49	−0.18
Fe^3+^-6H_2_O-FeS_2_-Sb_2_S_3_	Fe-S	2.271	0.48	−0.21

**Table 6:** Hirshfeld charge analysis of Sb in Sb_2_S_3_.

Atom	Charge/e Before adsorption	Charge/e Glucose adsorption	Charge/e Fe^3+^-6H_2_O adsorption
Sb	5.64	5.61	5.43

## 4 Conclusion

In this work, the effects of FeS_2_ on the bioleaching of Sb_2_S_3_ were investigated by combining experiments and DFT calculations, and the results can be summarized as follows: (1) After adding FeS_2_, the bioleaching rate of Sb_2_S_3_ increased significantly, from 2.23 to 24.6% after 5 days of bioleaching, and the best mass mixing ratio was 0.5:0.5; (2) During the bioleaching process, Sb_2_S_3_ was gradually transformed to Sb_2_O_3_ and Sb_2_O_5_; (3) Adding FeS_2_ can form a FeS_2_-Sb_2_S_3_ galvanic cell, which has a greater redox reaction current density, and faster electronic delivery efficiency; and (4) The DFT results indicated that after mixing, both Fe(III)-6H_2_O and glucose could adsorb onto the Sb_2_S_3_ (0 1 0) surface more easily, and Fe(III)-6H_2_O may play a major role in Sb_2_S_3_ bioleaching.

## Data Availability

The original contributions presented in the study are included in the article/Supplementary material, further inquiries can be directed to the corresponding authors.
