# Supplementary material for: The promotion effect of FeS2 on Sb2S3 bioleaching and Sb speciation transformation
Source: Front Microbiol. 2025 Jan 21;16:1475572. doi: 10.3389/fmicb.2025.1475572 (PMC11790673; doi:10.3389/fmicb.2025.1475572)
Supplement: Supplementary file 1 [file Data_Sheet_1.docx]

**Supporting information**

**The promotion effect of FeS_2_ on Sb_2_S_3_ bioleaching and Sb speciation transformation**

Xing-fu Zheng^1, 2, 3^, Jin-lan Xia^1, 3*^, Zhen-yuan Nie^1, 3^, Hong-peng Cao^1, 3^, Rui-Jia Hu^2^, Yu-ting Liang^1^. Hong-chang Liu^1, 3*^

^1^*School of Minerals Processing and Bioengineering, Central South University, Changsha 410083, China;*

*^2^ Guangxi Academy of Sciences, Nanning 530007, China;*

*^3^ Key Lab of Biometallurgy of Ministry of Education of China, Central South University, Changsha 410083, China;*

*****Correspondence to be addressed by e-mail: jlxia@csu.edu.cn; hchliu2050@csu.edu.cn.

**Fig. S1.** Sb extraction rates for sterile controls under varying amounts of FeS_2_ added.

**Fig. S2.** The curves for the total Fe (a) and Fe^2+^ (b) concentration for bioleaching of Sb_2_S_3_ under varying amounts of FeS_2_ added.


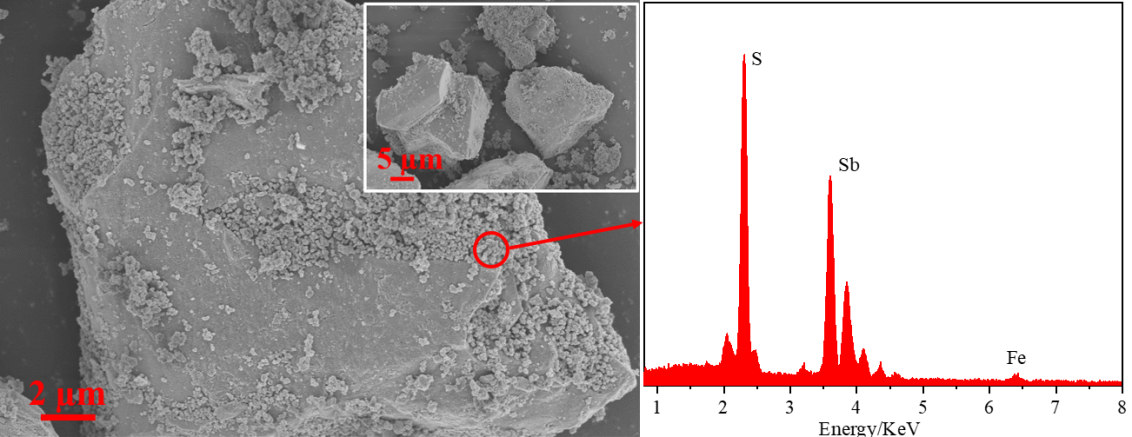


**Fig. S3** SEM images of the bioleaching residues for 10 days after adding FeS_2._


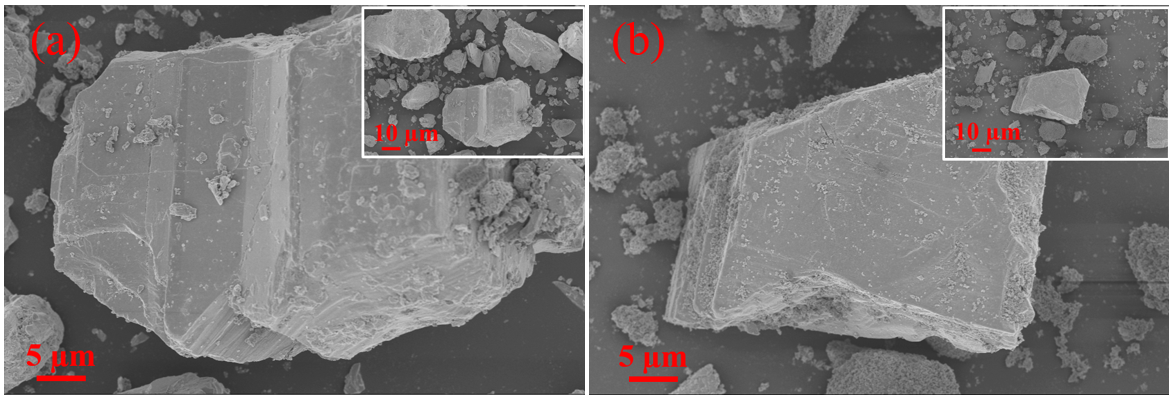


**Fig. S4** SEM images of the bioleaching residues without adding FeS_2_ for 0 days (a) and 10 days (b).

**Fig. S5** CV curves for FeS_2_-Sb_2_S_3_ with adding *S. thermosulfidooxidans*.

**Fig. S6** CV curves for Sb_2_S_3_ with adding Fe^3+^.


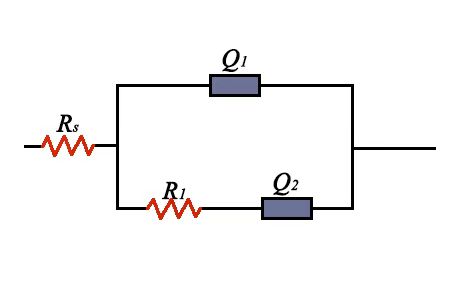


**Fig. S7.** Equivalent circuit diagram of EIS.


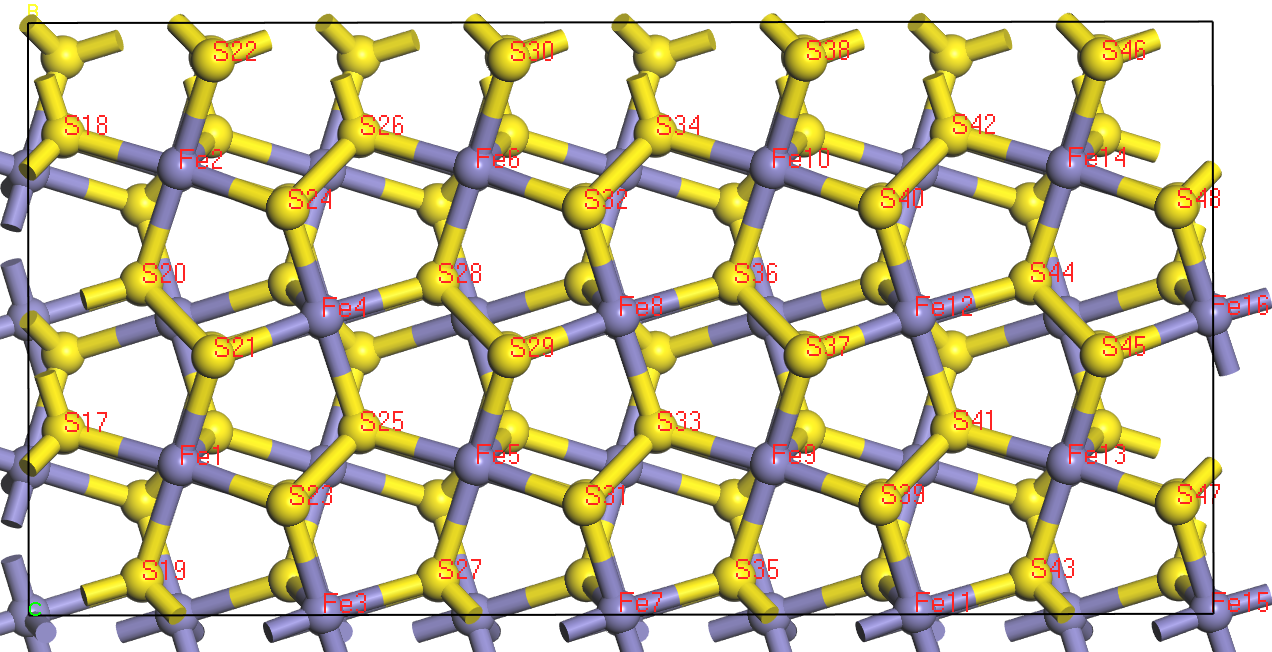


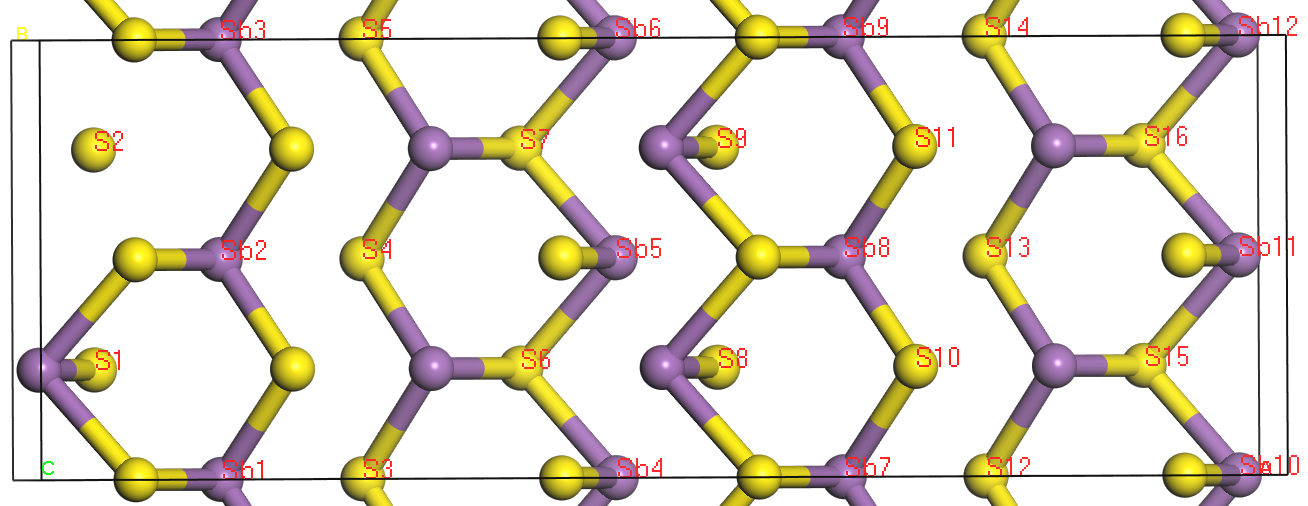


**Fig. S8.** (a) The upper surface of FeS_2_, bottom surfaces of Sb_2_S_3_.

Table S1 The XRF results of the main component (in the form of oxide) of stibnite

| Component | Content (%) |
| --- | --- |
| Sb_2_O_3_ | 37.66 |
| SO_3_ | 25.26 |
| SiO_2_ | 36.89 |
| Al_2_O_3_ | 0.28 |

Table S2 The ICP results of the Sb/S/Fe component of Sb_2_S_3_

| Component | Content (%) |
| --- | --- |
| Sb | 71.75 |
| S | 28.25 |
| Fe | 0 |
